# Semi-supervised adaptive-height snipping of the hierarchical clustering tree

**DOI:** 10.1186/s12859-014-0448-1

**Published:** 2015-01-16

**Authors:** Askar Obulkasim, Gerrit A Meijer, Mark A van de Wiel

**Affiliations:** Department of Epidemiology and Biostatistics, VU University Medical Center, Amsterdam, The Netherlands; Department of Pathology, VU University Medical Center, Amsterdam, The Netherlands; Department of Mathematics, VU University, Amsterdam, The Netherlands

## Abstract

**Background:**

In genomics, hierarchical clustering (HC) is a popular method for grouping similar samples based on a distance measure. HC algorithms do not actually create clusters, but compute a hierarchical representation of the data set. Usually, a fixed height on the HC tree is used, and each contiguous branch of samples below that height is considered a separate cluster. Due to the fixed-height cutting, those clusters may not unravel significant functional coherence hidden deeper in the tree. Besides that, most existing approaches do not make use of available clinical information to guide cluster extraction from the HC. Thus, the identified subgroups may be difficult to interpret in relation to that information.

**Results:**

We develop a novel framework for decomposing the HC tree into clusters by semi-supervised piecewise snipping. The framework, called guided piecewise snipping, utilizes both molecular data and clinical information to decompose the HC tree into clusters. It cuts the given HC tree at variable heights to find a partition (a set of non-overlapping clusters) which does not only represent a structure deemed to underlie the data from which HC tree is derived, but is also maximally consistent with the supplied clinical data. Moreover, the approach does not require the user to specify the number of clusters prior to the analysis. Extensive results on simulated and multiple medical data sets show that our approach consistently produces more meaningful clusters than the standard fixed-height cut and/or non-guided approaches.

**Conclusions:**

The guided piecewise snipping approach features several novelties and advantages over existing approaches. The proposed algorithm is generic, and can be combined with other algorithms that operate on detected clusters. This approach represents an advancement in several regards: (1) a piecewise tree snipping framework that efficiently extracts clusters by snipping the HC tree possibly at variable heights while preserving the HC tree structure; (2) a flexible implementation allowing a variety of data types for both building and snipping the HC tree, including patient follow-up data like survival as auxiliary information.

The data sets and R code are provided as supplementary files. The proposed method is available from Bioconductor as the R-package **HCsnip**.

**Electronic supplementary material:**

The online version of this article (doi:10.1186/s12859-014-0448-1) contains supplementary material, which is available to authorized users.

## Background

Hierarchical clustering (HC) is a popular data mining technique for detecting clusters of closely related objects in data, and is widely used in computational biology for the analysis of microarray data, DNA copy number data, phylogeny, and others. More than 90% of published clustering applications to microarray data use the HC [1]. Popularity of the HC is largely due to its robustness to the shape of clusters and the ability to represent nested clusters. The HC organizes objects into a hierarchical cluster tree (dendrogram) with branches that represent potential clusters. However, the HC algorithms do not actually partition the given data set into clusters, but only compute a hierarchical representation of the data. A HC tree derived from molecular data expresses the tendency of samples to be clustered by their molecular signatures. The HC has been used to identify clinically relevant cancer subtypes in several studies [2-5]. The focus of this study is placed on identifying clusters of patients that are located deep in the HC tree with distinct clinical outcomes. We achieve this goal by using high-dimensional molecular data augmented by additional clinical information.

Since there are no explicit clusters in the HC output, clusters are obtained either manually by visually inspecting the tree structure or by cutting the HC tree at a specific height, after which the resulting connected components are treated as clusters. The latter (referred as the fixed-height cut hereafter) is a simple, yet elegant technique commonly used in practice. However, extracting clusters via the fixed-height cut comes at a cost. As noted in several studies [4,6,7], it may fail to extract the nested clusters in the HC tree (see Additional file 1: Figure S1). Without losing the advantages the HC enjoys, alleviating the problems of the fixed-height cut approach is the focus of this paper. A second disadvantage of most unsupervised learning methods is that identified clusters (cancer molecular subtypes) are unrelated to patient clinical information [8]. Since the HC does not utilize the available clinical data, there is no guarantee that the identified subtypes will exhibit significant functional coherence.

We propose a novel procedure to extract clusters from the HC tree, called guided piecewise snipping. Our method resolves the drawbacks of the standard fixed-height cut approach by allowing the piecewise rather than the fixed-height cut, and incorporating available clinical data to decide upon the optimal cut. It snips the given HC tree (possibly) at different heights to ensure a) a high score for cluster homogeneity; and b) a high score for consistency with the clinical data used for guidance. Hence, the optimal partition is the one ranked high by both data types. Note that the co-use of clinical data for evaluating a partition helps to make the clustering more robust, a major concern when dealing with high-dimensional molecular data.

**Figure 1:**
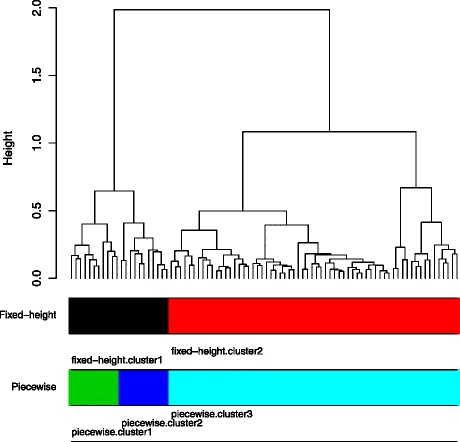
**The HC tree corresponds to the Lung.1 data set, and the optimal cuts induced by the two approaches.** The fixed-height cut approach produces the the HC tree into two clusters (*fixed-height.cluster1-2*). The piecewise snipping, on the other hand, renders three clusters by splitting the *fixed-height.cluster1* into two sub-clusters (*piecewise.cluster1-2*).

**Figure 2:**
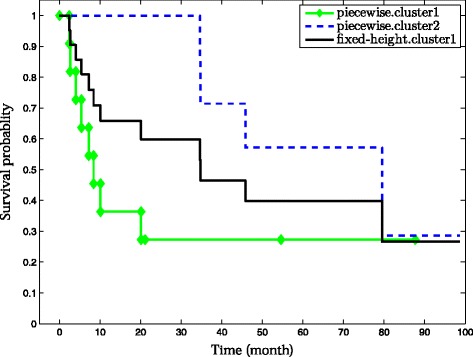
**Survival curves correspond to the clusters formed on the left branch of the HC tree in Figure** 1 **.**

Let us now illustrate some of the drawbacks of, first, fixed-height cuts and, second, unguided clustering evaluation. We use the Lung.1 gene expression data set [5] (Table 1) for which patient follow-up information is also available. A HC tree with *Pearson* correlation as similarity metric and *Ward* [9] linkage is derived using the expression data (Figure 1). The complete set of partitions, where a partition (clustering) is composed of non-overlapping clusters, is extracted by cutting (with a straight line) the HC tree at all possible heights. To assess the quality of a partition, the Cox model is used to quantify the association between cluster labels in the partition and the patient survival time. Then, the likelihood and the Akaike information criterion (AIC) are obtained. The optimal partition induced by the fixed-height cut (AIC=177) is composed of two clusters (*s.cluster1-2*). For illustration purposes, we keep the *s.cluster2* fixed, and decompose the *s.cluster1* (by piecewise snipping) into two sub-clusters *p.cluster1-2*. The new partition decreases the AIC to 166. In addition, patients in the *p.cluster1-2* manifest very different survival outcomes (logrank test *p*-value = 0.04). This shows that there are clinically heterogeneous clusters on the left branch of the HC left branch of the HC tree that are forced to merge by the fixed-height cut (Figure 2). A simulation study also confirms the limitations of the fixed-height cut (see further on and the Additional file 1).

**Table 1:** **Data set summaries**

**Cancer type**	**Reference**	**# Samples (feat.)**	**Data type**
Lung.1	*Beer(2002)*	86(7129)	mRNA
Leukemia	*Bullinger(2004)*	116(6283)	mRNA
Lung.2	*Bhattacharjee(2001)*	125(3171)	mRNA
Lymphoma	*Rosenwald(2002)*	240(7399)	mRNA
Prostate	*Sboner(2010)*	281(6100)	mRNA
Glioblastoma	*Verhaak(2010)*	158(10850)	DNA

Time-to-death follow-up information is available for all data sets except for the Leukemia data set for which follow-up data is time-to-relapse.

Next, we apply one of the well-known *unguided* clustering algorithms, Dynamic Tree Cut [6] to the HC tree in Figure 1. The algorithm decomposes the HC tree into 13 clusters (Additional file 1: Figure S2) that renders *AIC*=204. Clearly, lack of guidance leads to unnecessary splits (merges) of the clusters. Often, clinical data is also available, and may be used to “guide” or “adjust” the process of clustering.

Semi-supervised clustering approaches to integrate prior biological knowledge into the clustering procedure have added much to endeavor [10,11]. Many algorithms have been proposed to exploit the domain knowledge and to improve cluster relevance, with significant improvements over their unsupervised counterparts [8,12]. Some studies used model-based integration approaches to explicitly model the joint probability of gene expression and functional labels of genes. Other studies proposed to modify the distance between expression profiles prior to the HC tree construction. These modifications usually produce different clustering results from the ones produced solely on the basis of the samples’ molecular profiles. To our knowledge, so far only two approaches have been proposed to snip the HC tree at variable heights. One is a HC tree snipping method specific for gene clustering only. It snips the tree branches at different heights to induce clusters that are maximally consistent with partially available gene labels [10]. Another one is a semi-supervised approach, called VI-cut, that has been proposed to decompose a HC tree into clusters that optimally match a set of known annotations [13]. These approaches are, however, not without problems. For example, the former is designed for gene clustering; application to sample clustering application is not straightforward, because “clustering samples is very different to clustering genes” [14]. The second one only allows gene expression data. Besides that, both require the auxiliary information in discrete format, i.e. class labels of the samples, which limits these methods’ utilities in fully exploiting the HC tree structure. It is desirable to have a procedure that is applicable to a wide variety of data types. Continuous-scaled patient clinical data, such as survival times, is often available [2,3]. One could discretize these, but this comes at a cost of losing information [15].

Guided piecewise snipping enjoys the following three advantages over existing approaches. First, any type of clinical data can be used to “guide” the tree snipping process, no discretization is needed. Second, the optimal partition will be selected on the basis of its quality in both data spaces, which makes the result more stable and easy to interpret. Third, our algorithm uniquely applies a molecular to follow-up projection principle when assigning test samples (for which the follow-up data is unknown) to one of the existing clusters. While the guided piecewise snipping approach proposed in this paper can accept many types of input, we focus in our examples on the case where the given two data types are high-dimensional molecular data and (possibly censored) time-to-event data (i.e. survival, remission, tumor recurrence) throughout (see Discussion and conclusion).

## Results

Performance comparisons of the two approaches are conducted using multiple medical data sets. Before presenting experimental results, we first summarize these data sets. Additional results are given in the Additional file 1.

### Data

A summary of five publicly available gene expression data sets with time-to-event information used in this study is given in Table 1. To show the generality, a DNA copy number data set is also included.

### Comparison

For the data sets above, performance of the guided piecewise snipping was compared with a)unguided piecewise snipping, which makes no use of survival data; and b) guided and unguided fixed-height cuts. The unguided procedures use only WSS as evaluation criterion on the clustering. In addition, we assess whether test label assignment to the clusters by our novel PNNC-distance improves assignment based on by *Ward*-distance. The quality of the partitions is assessed by the statistical testing procedure described above. We report the median log-rank *p*-value over the three folds of the cross-validation in Table 2. In addition, we report the global test [16] *p*-value, which quantifies the overall association between the molecular and time-to-event data in the entire data set. If such a *p*-value is small, one expects a more beneficial effect of guidance than when it is large.

**Table 2:** **Comparison of a) unguided fixed-height cuts; b) guided fixed-height cuts; c) unguided piecewise snipping; d) guided piecewise snipping; e) guided piecewise snipping + PNNC**

**Data**	**Fixed-height cut**			**Piecewise snipping**		
	**Unguided**	**Guided**	**Unguided**	**Guided**	**Guided**	
	**Ward**	**Ward**	**Ward**	**Ward**	**PNNC**	**Global test**
Lung.1	0.394	0.167	0.308	0.071	0.016	0.019
Leukemia	0.688	0.238	0.336	0.016	0.002	0.004
Lung.2	0.256	0.497	0.418	0.103	0.048	0.227
Lymphoma	0.149	0.113	0.176	0.038	0.001	0.001
Prostate	0.086	0.360	0.091	0.083	0.107	0.002
Glioblastoma	0.142	0.160	0.064	0.041	0.103	0.003

All guided approaches use *WSS + AIC* as cluster evaluation criterion, the unguided approaches use WSS only; a)-d) use *Ward* distance for test sample assignment, whereas e) uses PNNC. The first five numeric columns contain the median log-rank *p*-values across the three splits of the 3-fold CV. The last column contains the *p*-values from the global test for overall association between the molecular and time-to-event data.

From Table 2 it is clear that in most of the data sets the piecewise snipping approaches outperform their fixed-height cut counterparts, in particular when guidance is used. In addition, the guided piecewise snipping clearly outperforms the unguided piecewise snipping when the global test indicates a strong association between the two data types. Finally, the test label assignment by the novel PNNC-distance results in a smaller log-rank *p*-value than the corresponding one of the assignment by *Ward* for 4/6 data sets, indicating the potential benefit of PNNC.

### Visualization

To assist in visual inspection of the differences between the clusters induced by the two approaches, we summarize clusters in terms of 1) time-to-event information; patients’ follow-up information in each cluster is used to construct the Kaplan-Meier survival curve. 2) molecular profiles of the samples; summarized by molecular entropy, as a measure of overall expressions [17]. Here, we present the results for the Leukemia data, using the *BIC*+*GK* criterion for partition evaluation.

**Figure 3:**
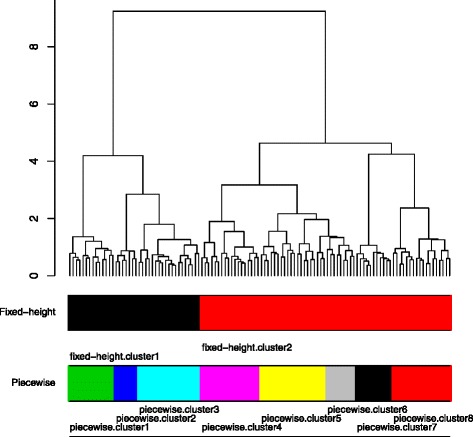
**The HC tree corresponds to the Leukemia data set, and the optimal cuts induced by the two approaches.** While, the fixed-height cut decomposes the HC tree into two big clusters (*fixed-height.cluster1-2*), eight clusters (*piecewise.cluster1-8*) are produced by the piecewise snipping.

**Figure 4:**
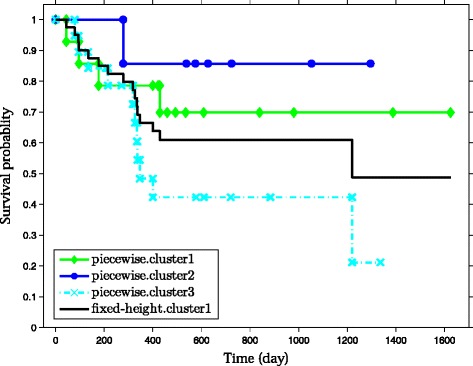
**The survival curves correspond to clusters on the left branch of the HC tree in Figure** 3 **.**

Figure 3 shows the derived HC tree, and the cuts induced by the two approaches. The fixed-height cut approach generates a partition with two clusters (*s.cluster*), while the guided piecewise snipping identifies six clusters (*p.cluster*). The latter splits the two big clusters (*s.cluster1-2*) into sub-clusters with different sizes. The survival curves associated with the *s.cluster1* and *p.cluster1-3* on the left branch of the HC tree are shown in Figure 4. The three sub-clusters turn out to contain patients with relatively different clinical outcomes (*p*-value = 0.1^1^). Cluster *p.cluster1* corresponds to the medium good-prognosis group with 29% patients (4/14) who experienced relapse, *p.cluster2* corresponds to the good-prognosis group containing 14% (1/7) relapsed patients, and *p.cluster3* corresponds to the poor-prognosis group in which 58% (11/19) of patients experienced relapse of the tumor. Violin plots of cluster entropies (Additional file 1: Figure S7) further show that molecular profiles are also different in these three clusters.

**Figure 5:**
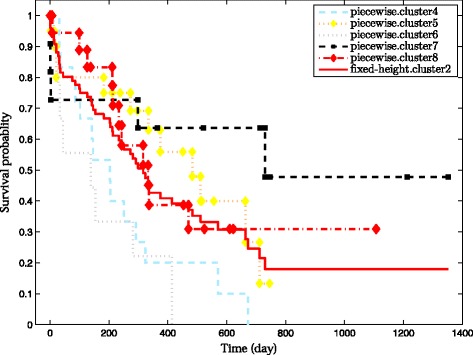
**The survival curves correspond to the clusters on the right branch of the HC tree in Figure** 3 **.**

The same phenomenon is observed on the right branch of the HC tree. The standard approach again merges all patients in this branch into one cluster, but five sub-clusters are identified by our approach. Observe in Figure 5 that, these five clusters are also manifest clinically different characteristics (*p*-value = 0.05 ^*a*^). In particular, note *p.cluster6*; it is hidden rather deeply in the tree, but has the worst survival, much worse than its neighbors e.g. *p.cluster5*. Entropy distributions in these clusters are also support this observed differences (see Additional file 1: Figure S7). Especially, the average entropy of *p.cluster6* is considerably higher than the rest.

We also examine the association between the optimal partitions found by the two approaches in the Lung.1 data set and that of the clinical outcomes, Differentiation and Stage, not used in the cluster extraction process. In particular, Stage has stronger association with the guided piecewise snipping induced partition than the one from the fixed-height cut, which shows that the former is potentially also more relevant for outcomes other than survival (see Table S3).

Illustrations for the other data sets are given in the Additional file 1. While for most of the data sets guided piecewise snipping renders clusters substantially different from those from the fixed-height cut, there are some cases in which two approaches produce similar results (see Additional file 1: Figure S15).

### Simulation results

We also performed a simulation study, which serves to illustrate the effect of cluster heterogeneity on the performance of both fixed-height cutting and piecewise snipping and to compare the two. In this simulation we do not involve follow-up information, because the potential use of clinical follow-up to improve the cluster results was already demonstrated for several real data sets (see Table 2). In addition, follow-up information is not necessary to demonstrate that, as opposed to piecewise snipping the fixed-height cut may sometimes not capture true clusters (known from the simulation setting) that are hidden deeply in the HC tree.

The Additional file 1 contains a complete description of the simulation and its results. Here, we provide a summary. We studied three scenarios. Expression data are simulated from a multivariate Normal mixture distribution, where the mixture components represent the clusters. The overlap between the components quantifies the cluster heterogeneity: the larger the overlap, the more difficult the extraction of the clusters from the HC tree that is generated from the simulated data. For three scenarios with increasing cluster heterogeneity Additional file 1: Figures S2 to S4 show the resulting HC trees and the true cluster labels of each of the simulated samples. The fixed-height cut and the piecewise snipping were applied to the HC tree using WSS as evaluation criterion. The two algorithms are compared by the Adjusted Rand Index (ARI), which is a standard metric for measuring the overlap between the true clustering and the data-driven one.

The simulation study shows that if the true clusters are homogeneous and well-separated (Scenario 1), both fixed-height cutting and piecewise snipping perform well (Additional file 1: Figure S2). Still the ARI of piecewise snipping is somewhat better (1 vs 0.93), because it better discerns true cluster 4 from 3. For scenario 2, with intermediate level of cluster heterogeneity, piecewise snipping clearly outperforms fixed-height cutting, ARI = 0.46 vs ARI = 0.27, in particularly because it captures the 4-cluster deeper in the HC tree (Additional file 1: Figure S3). Naturally, when the clusters are very heterogenous, as in scenario 3, both methods perform poorly, which is a consequence of the HC tree not being able to group samples from the same true clusters (Additional file 1: Figure S4). Yet, piecewise snipping performs somewhat better (ARI = 0.12 vs ARI = 0.09). Hence, we conclude that the cost of generating the complete search space for piecewise snipping as opposed to the limited search space for fixed-height cut is definitely offset by the superior performance of the former.

## Discussion and conclusion

We introduced a novel HC tree snipping method called guided piecewise snipping, which uses molecular data and available follow-up information to induce optimal snipping point(s) in the HC tree. Guided piecewise snipping features a number of novelties and advantages when compared to existing approaches. First, unlike other piecewise snipping methods our approach is able to deal with (possibly censored) time-to-event data like survival or relapse. Second, our bivariate rather than commonly used univariate evaluation of partitions forces the resulting clusters to be both stable in the molecular data space and associative in the clinical response space. Third, we demonstrated the superiority of semi-supervised piecewise snipping over more conventional semi-supervised fixed-height cuts in terms of significant associations of (sub-)clusters with follow-up that were not detected by the fixed-height cut. Note that even when the piecewise snipping renders the same result as the fixed-height cut, our approach is useful in the sense that it objectively decides where to snip. Finally, we introduced *PNN+Concordance* as a new test-sample cluster assignment. We showed superior performance with respect to Ward-linkage-based assignment when the association between the molecular data and clinical response is strong. Note that the latter is the relevant situation for our method: if this association is not significant then the snipping may not be effective. Hence, it may be useful to apply the global test [16] as a pre-test to decide about using supervised snipping or not.

We emphasize that our ambition is to find *meaningful* clusters rather than *true* clusters. In general ‘true clusters’ are unlikely to exist as such: at best, clusters are a discrete approximation of a more continuous truth. How ‘true’ a partition is, depends on the context: for treatment options two clusters may be very different, whereas from a survival perspective they may be very similar. Also, note that current subtypes are always based on particular choices of the distance metric and linkage method used; it is well-known that other choices would have lead to different partitions, and hence different subtypes.

The guided piecewise snipping differs fundamentally from supervised prediction (survival prediction, classification) methods, in its approach, in its goal and in its application. First of all, the roles of molecular and time-to-event data are reversed: supervised prediction centers around the latter and uses the molecular to issue a prediction; our approach uses time-to-event data to guide ‘prediction’ of molecular subgroups. In addition, unlike many supervised prediction methods, our approach is fundamentally non-linear in the molecular space. Our aim is similar to conventional unsupervised clustering: find good subgroups of patients rather than a good overall prediction of time-to-event follow-up. Since the cluster extraction process is guided by time-to-event data, we may isolate subgroups that are rather deep in the HC tree, so the molecular differences with their neighbours are subtle but apparently relevant for, f.e., survival (Additional file 1: Figure S28). Finally, note that, unlike the results of supervised prediction, our result, the subgroups, may assist in better understanding of the molecular taxonomy of the disease under study.

There are a number of extensions that may be incorporated in future versions of this methodology. For example, from a HC tree one may obtain the height information at which two clusters are merged. A small incremental value denotes that there is strong evidence in the data that two clusters are similar. Incorporating this information into the partition evaluation process by giving higher scores to partitions in which clusters are composed of samples that are merged at comparatively lower height differences is a interesting line of research. Throughout this study, we use patient time-to-event follow-up data as clinical data to identify biologically and clinically meaningful clusters. However, other clinical variables, such as the stage of tumor, tumor remission status and metastasis information etc., may also be used. One only needs to adapt the cluster quality measure suitable for the data at hand. For example, suppose we are given binary tumour remission status and wish to identify a partition in which clusters are strongly associated with remission status. In this case, one may use the in-group proportion criteria [18] to select the optimal partition.

## Methods

**Figure 6:**
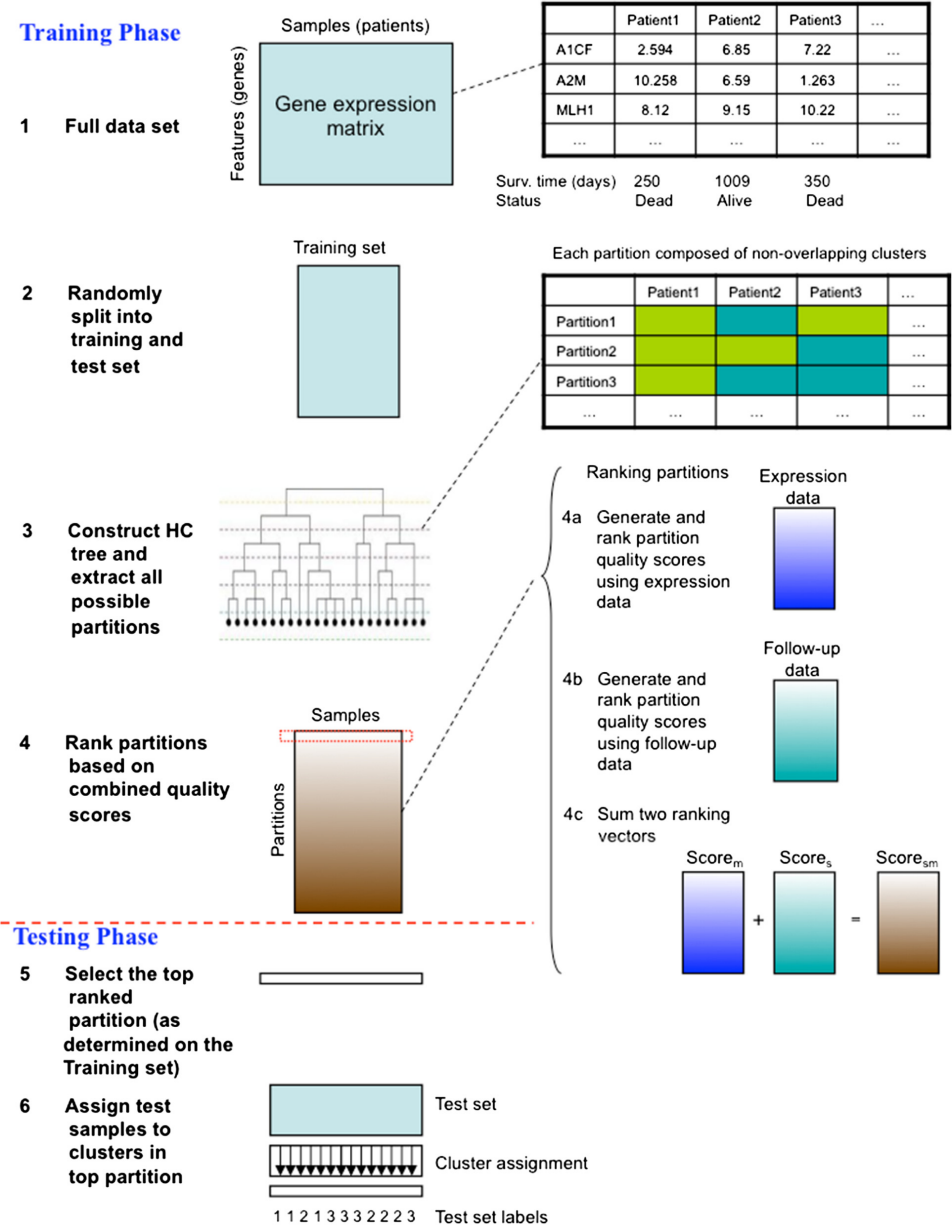
**The work flow of the piecewise snipping approach.**

Our guided piecewise snipping approach is composed of three steps to tease out meaningful clusters from a given HC tree (Figure 6). In this section, we present a brief description of each step, which also provides the partial backdrop of our motivations for this work. More details, also on the use of the algorithm, are given in the Additional file 1.

### The HC tree construction

While our method applies to any distance metric and linkage method, we base all examples on the *Pearson* correlation as a distance measure and *Ward’s* minimum variance as a linkage measure to avoid tuning of the results. An exception is the Glioblastoma data set (DNA copy number) for which the distance matrix is calculated by the dedicated R-package **WECCA** [19] with parameters “ordinal” and “heterogeneity”. We assume that the given HC tree well expresses the similarity between samples in terms of their molecular profiles. Applying feature selection may improve the quality of the tree, but to avoid bias to a particular feature selection technique, we used a full set of features in all data sets. The evaluation of the optimality of the particular distance metric or linkage method that optimal for the data set at hand is beyond the scope of this study.

### The search space construction

For guided piecewise snipping, the search space is composed of all possible partitions that can be extracted from the given HC tree. The size of the search space depends on the number of samples in the data and the level of skewness of the HC tree. A balanced tree produces more partitions than a skewed one. We develop an efficient algorithm to extract the complete set of partitions from a HC tree in acceptable time (see the Additional file 1). For example, the largest data set we used in our analysis is the Prostate cancer data set [7] (Table 1), which consists of measurements from 281 patients. Our algorithm took about three minutes to extract 70910 possible partitions on a normal dual core CPU 3.16GHz, 4GB of RAM desktop computer. To reduce the effect of outliers, and ensure that the quality of clusters is reliably estimated in a later stage, we set a threshold on the minimum number of samples (set to 4 in all data sets) in each cluster. For a very large data set, this is beneficial as it obviates the need to a assess large number of trivial partitions (which encompass too many tiny clusters). Particularly, if one has prior knowledge of an (approximate) upper (or lower) bound for the cluster size, the constraint placed on the size of clusters may exclude partitions that deviate too much from one’s expectation. Extensive experiments with real-world data sets show that the minimum cluster size in the optimal partition is often not bounded by the threshold.

For the fixed-height cut, the search space is much smaller: it consists only of those partitions that results from cutting the HC at a fixed height. This constitutes the main difference between the fixed-height cut and piecewise snipping.

### Partition evaluation

The aim of clustering is to uncover meaningful groups in the data. However, not any arbitrary partitioning of a given data set reflects such structure. Upon obtaining the complete set of partitions from a given HC tree, the next step is to evaluate each partition objectively and select the optimal one among a large number of candidates. The selected partition should unravel the hidden data structure and should be stable enough to extrapolate to new samples. A cluster quality measure is a function that, given a data set and its partition into clusters, returns a real number representing how strong or conclusive the clustering is. Existing approaches allow for evaluating a partition either using molecular data alone, or only using follow-up information (which is required to be in discrete format). In order to obtain a robust clustering and represent the data structure in both data spaces, we propose the following strategy, depicted in Figure 6:

1. Evaluate partitions using molecular data alone (call it score_*m*_). Given the number of cluster quality measures exist, it is infeasible to explore all in our application. Refer to [20] for a overview of existing cluster quality measures. In this work we mainly focus on the Within-cluster-Sum-of-Square (WSS); results for alternative measures like the C-index and Goodman-Kruskal index (GK) were qualitatively very similar. For a given partition and corresponding distance matrix, we calculate an overall quality score using one of these measures.
2. Evaluate partitions using follow-up data alone (call it score_*s*_). Here, we use the cluster labels in each partition as a covariate and time-to-event data as response variable to assess their association. We apply a measure tailored to time-to-event task: modified AIC [21]. Unlike *p*-values, this criterion allows comparison across partitions that include different number of clusters. Qualitatively, use of ordinary AIC and (modified) BIC [22] do often not lead to considerable differences.
3. Rank score_*m*_ and score_*s*_ across partitions from good to bad, separately. The final evaluation score of each partition is the summation of the two ranking values (call it score_*ms*_). A partition with the smallest score_*ms*_ is selected as the optimal clustering. Our motivation to use ranks rather than score_*m*_ and score_*s*_ directly is that the latter two are on very different scales, whereas the ranks are on the same scale, thus suitable for adding.

The criteria above also apply to partitions created by *guided* fixed-height cutting, hence this differs from guided piecewise snipping only by the size of the search space. The illustrations in the Results section show that the superior performance of our approach is not an artifact of the larger search space induced by piecewise snipping: the superior performance remains when evaluated on independent test samples on a variety of cancer molecular data sets. Note that the choice of cluster quality measures and information criteria is free. In our flexible implementation one can directly insert the alternatives mentioned above and ones own metrics as well.

### Clustering on the test set

To estimate and compare performances of the guided piecewise snipping and the fixed-height cut approaches in an unbiased manner, we perform 3-fold cross-validation (3CV) to split the data three times into non-overlapping training and test sets, such that each sample belongs to the test set exactly once. For a given split, an optimal partition is derived using the training set, then each test sample is assigned to one of the clusters in the optimal partition. Note that, for the test set, we assume that the patient time-to-event information is not available. If the clustering found in the training phase is “good” enough to represent the hidden data structure, then we expect relatively high-quality clustering on the test set.

The *Ward* distance metric used to construct in the HC tree, may also be used to assign cluster labels of the optimal partition found in the training phase to the test samples. But, we have to keep in mind that, the nature of the purposed approach here is semi-supervised clustering. The *Ward* distance simply assigns a test sample to a particular cluster based only on the similarity of their molecular profiles. However, due to noisy nature of molecular data, the observed molecular profile of a sample may appear to be more similar to members of a cluster that differ considerably in terms of their time-to-event information. Like in the training phase, clustering on the test set should, ideally, also take into account the similarities between samples both in the molecular and follow-up data spaces. Since we assume that the latter is not available for the test set, we decide to make use of a novel distance metric based on an indirect projection principle, termed *PNN+Concordance* [23].

The *PNN+Concordance* (PNNC) approach works as follows: first, it determines a test sample’s nearest neighbours (NN) in the molecular data space of the training set, subsequently the pseudo nearest neighbours [23] (PNN) in the time-to-event data space. The PNN of the test sample are determined by considering the projection of the NNs onto the time-to-event space, as well as the neighbours of the NN in this space. PNNC uses Harrel’s concordance index [24] to decide the similarity between PNN and members of a cluster under consideration. For the sake of comparison, we present the results from both *Ward* and PNNC. A detailed description of the *PNN+Concordance* approach, including a toy example, is given in the Additional file 1.

### Validation

We validate the two approaches using a statistical testing perspective. For a given split of the data, the testing perspective is simple: we assign test samples to the clusters (as created by either the guided piecewise snipping or the fixed-height cut) and test the null-hypothesis that the clusters to which the test subjects are assigned are independent of the actual survival times of those subjects. To this end, we apply a log-rank test. For each of the three splits (when using 3CV), the test renders one *p*-value that represents how well the clustering resulting from the training data discriminates (new) samples with respect to the time-to-event (survival) data. Note that the resulting *p*-values are not independent due to the overlap between training samples in one split and test samples in another. The median of the three *p*-values, however, is a valid measure of significance [25].

## Availability and requirements

- Project name: HCsnip
- Project home page: http://www.bioconductor.org/packages/release/bioc/html/HCsnip.html
- Operating system(s): Platform independent
- Programming language: R
- Licence: General Public Licence (≥2)

## Endnotes

^1^ Bias adjusted *p*-value obtained by permutation. See the Additional file 1.

## Additional file

Additional file 1
**Supplementary Document.** This document provides the detailed description of the piecewise snipping algorithm, and some results not included in the paper.

## References

[1] de Souto M, Costa IG, de Araujo D, Ludermir TB, Schliep A (2008). Clustering cancer gene expression data: a comparative study. BMC Bioinformatics.

[2] Alizadeh AA, Eisen MB, Davis RE, Ma C, Lossos IS, Rosenwald A, Boldrick JC, Sabet H, Tran T, Yu X, Powell JI, Yang L, Marti GE, Moore T, Hudson JJ, Lu L, Lewis DB, Tibshirani R, Sherlock G, Chan WC, Greiner TC, Weisenburger DD, Armitage JO, Warnke R, Levy R, Wilson W, Grever MR, Byrd JC, Botstein D, Brown PO, Staudt LM (2000). Distinct types of diffuse large B-cell lymphoma identified by gene expression profiling. Nature.

[3] Bhattacharjee A, Richards WG, Staunton J, Li C, Monti S, Vasa P, Ladd C, Beheshti J, Bueno R, Gillette M, Loda M, Weber G, Mark EJ, Lander ES, Wong W, Johnson BE, Golub TR, Sugarbaker DJ MM (2001). Classification of human lung carcinomas by mRNA expression profiling reveals distinct adenocarcinoma subclasses. Proc Natl Acad Sci USA.

[4] Sørlie T, Perou CM, Tibshirani R, Aas T, Geisler S, Johnsen H, Hastie T, Eisen MB, van de Rijn, Jeffrey SS, Thorsen T, Quist H, Matese JC, Brown PO, Botstein D, Lønning PE, Børresen-Dale AL (2001). Gene expression patterns of breast carcinomas distinguish tumor subclasses with clinical implications. Proc Natl Acad Sci USA.

[5] Beer DG, Kardia SLR, Huang CC, Giordano TJ, Levin AM, Misek DE, Lin L, Chen G, Gharib TG, Thomas DG, Lizyness ML, Kuick R, Hayasaka S, Taylor JMG, Iannettoni MD, Orringer MB (2002). Gene-expression profiles predict survival of patients with lung adenocarcinoma. Nature.

[6] Langfelder P, Zhang B, Horvath S (2008). Defining clusters from a hierarchical cluster tree: the Dynamic Tree Cut package for R. Bioinformatics.

[7] Sboner A, Demichelis F, Calza S, Pawitan Y, Setlur SR, Hoshida Y, Perner S, Adami HO, Fall K, Mucci LA, Stampfer M, Andersson SO, Varenhorst E, Gerstein MB, Golub TR, Rubin MA, Andrén O, Kantoff11 WP, Johansson10 JE (2010). Molecular sampling of prostate cancer: a dilemma for predicting disease progression. BMC Med Genomics.

[8] Bair E, R T (2004). Semi-supervised methods to predict patient survival from gene expression data. PLoS Biol.

[9] Ward JHJ (1963). Hierarchical grouping to optimize an objective function. J Am Statist Assoc.

[10] Dotan-Cohen D, Melkman AA, Kasif S (2007). Hierarchical tree snipping clustering guided by prior knowledge. Bioinformatics.

[11] Kustra R, Zagdański A. Incorporating gene ontology in clustering gene expression data. In: CBMS ’06 Proceedings of the 19th IEEE Symposium on Computer-Based Medical Systems Salt Lake City, UT. IEEE: 2006. p. 555–563.

[12] Ideker T, Dutkowski J, L H (2011). Boosting signal-to-noise in complex biology: prior knowledge is power. Cell.

[13] Navlakha S, White J, Nagarajan N, Pop M, Kingsford C (2010). Finding biologically accurate clusterings in hierarchical tree decompositions using the variation of information. J Comp Biol.

[14] Granitto PM, Bayá AE (2011). Clustering gene expression data with a penalized graph-based metric. BMC Bioinformatics.

[15] Royston P, Altman DG, Sauerbrei W (2006). Dichotomizing continuous predictors in multiple regression: a bad idea. Stat Med.

[16] Goeman JJ, van de Geer, De Kort F, van Houwelingen HC (2004). A global test for groups of genes: testing association with a clinical outcome. Bioinformatics.

[17] van Wieringen WN, van der Vaart AW (2010). Statistical analysis of the cancer cells molecular entropy using high-throughput data. Bioinformatics.

[18] Kapp AV, Tibshirani R (2007). Are clusters found in one dataset present in another dataset?. Biostat.

[19] van Wieringen WN, van de Wiel, Ylstra B (2008). Weighted clustering of array CGH data. Biostat.

[20] Milligan GW (1981). A Monte-Carlo study of 30 internal criterion measures for cluster-analysis. Psychometrica.

[21] Liang H, Zou G (2008). Improved AIC selection strategy for survival analysis. Comput Stat Anal.

[22] Volinsky TC, E RA (2000). Baysian information criteria for censored survival models. Biometrics.

[23] Obulkasim A, Meijer GA, van de Wiel (2011). Stepwise classification of cancer samples using clinical and molecular data. BMC Bioinformatics.

[24] Harrell FEJ, Califf RM, Pryor DB, Lee KL, Rosati RA (1982). Evaluating the yield of medical tests. JAMA.

[25] Van de Wiel, Berkhof J, van Wieringen WN (2009). Testing the prediction error difference between 2 predictors. Biostatistics.

