# Supplementary material for: Semi-supervised adaptive-height snipping of the hierarchical clustering tree
Source: BMC Bioinformatics. 2015 Jan 16;16(1):15. doi: 10.1186/s12859-014-0448-1 (PMC4302100; doi:10.1186/s12859-014-0448-1)
Supplement: Additional file 1 — Supplementary Document. This document provides the detailed description of the piecewise snipping algorithm, and some results not included in the paper. [file 12859_2014_448_MOESM1_ESM.pdf]

Semi-supervised adaptive-height snipping of the hierarchical clustering tree

Supplementary document

Askar Obulkasim[1,*], Gerrit A. Meijer[2] and Mark A. van de Wiel[1,3]

[1]Department of Epidemiology and Biostatistics VU University Medical Center

[2]Department of Pathology, VU University Medical Center

[3]Department of Mathematics, VU University, Amsterdam, The Netherlands

This document provides supplementary information not mentioned in the paper.

Motivational Example

Sørlie *et al.,* (2001) used gene expression data to cluster the breast carcinomas using hierarchical clustering, then correlated the extracted clusters to that of clinical outcomes. They reported six clusters, each of them with unique clinical characteristics (Additional file 1: Figure S1). After closely examining the HC tree we find that, the reported six clusters cannot be extracted by cutting with a straight line at any place. At most, five out of the six clusters (solid line) can be retrieved, unless a piecewise snipping is placed in the left branch (broken line). Observe that, albeit their differences in terms of molecular (i.e. TP53 status) and clinical (i.e. survival time) characteristics, the Basel-like and the ERBB2+ tumours were merged by the the fixed-height cut approach. As this real life example shows, the fixed-height cut, in some occasions, may produce a sub-optimal result. This is because sometimes informative clusters in a branch of the HC tree may be located at a deeper level than the other branches.

Performance comparison between the piecewise snipping and the fixed-height cut approaches using simulated data sets

Our paper presents two novelties: a) we allow detection of clusters in the HC-tree to be guided by clinical response, such as survival; b) we introduce piecewise snipping as opposed to fixed-height cutting to be able to detect clusters deeper in the HC-tree. With the following simulations we aim to give more insight into b). We do not involve follow-up information here, because 1) It is not necessary for demonstrating b); 2) It has already clearly be shown for real data that inclusion of follow-up to guide the snipping can be beneficial (see Table 2 in the Main Document); and c) it is difficult to simulate survival

*To whom correspondence should be addressed. askar703@gmail.com

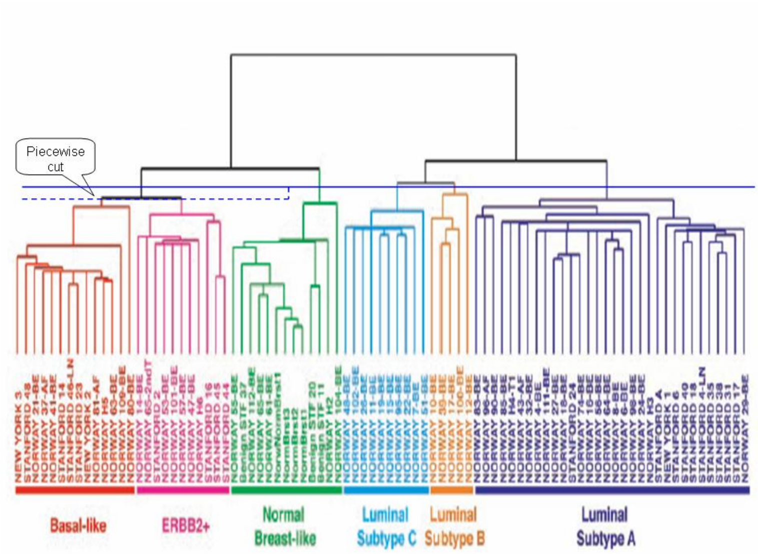

Additional file 1: Figure S1. The piecewise snipping versus the straight-line cut.

data corresponding to the clusters in an *objective* way. Hence, we simply use within-sum-of-squares (WSS) to evaluate the clusterings here.

We compared performances of the two approaches on simulated data sets from 200-dimensional Gaussian mixtures with six heterogeneous non-spherical covariance matrices (representing the true clusters) according to three levels of pairwise overlap between mixtures. For each level of overlap, 100 mixtures were simulated, then a data set of 50 observation is generated from each mixture. Subsequently, a HC tree is constructed with Pearson correlation as a distance and Ward linkage; the search space is obtained and the best partition is selected for each method separately. The performance of methods is evaluated using the Adjusted Rand Index (ARI), i.e. the original true cluster labels and the optimal partition obtained from each method are compared and the level of agreement is quantified. This simulation setting has been used by Melnykov *et al.,* (2012) to evaluate many well-known clustering algorithms. This simulation study was performed using the R-package MixSim (Melnykov *et al.,* 2012).

As mentioned in the paper piecewise snipping performs superior when nested clusters are present in the HC tree. Due to inhomogeneity of the clusters, some clusters may appear quite deep in one branch of the HC tree compared to other branch(s). Since the piecewise snipping searches the space composed of all possible partitions, the hidden clusters are highly likely to be detected. On the other hand, notwithstanding its popularity, because of its 'cut all branches at the same height' rule the fixed-height cutting fails to discover these clusters. To compare the two competing approaches, we present three scenarios where a HC tree has a) nested clusters with almost no overlap between clusters; b) the scenario where a HC tree has some overlapping nested clusters (heterogeneous clusters) and the scenario where a HC tree has highly overlapping clusters (highly heterogeneous clusters).

- The scenario where a HC tree has highly homogenous, non-overlapping nested clusters. This scenario was realized by setting the average pairwise cluster mixture proportions to 0.001 (parameter *BarOmega* in the *MixSim*).

  The HC tree in Additional file 1: Figure S2 shows that both approaches perform

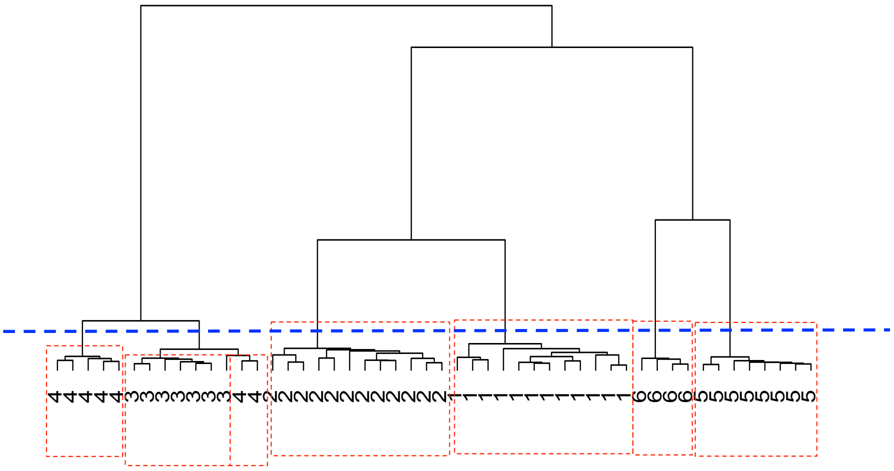

Additional file 1: Figure S2. A simulated HC tree with non-overlapping nested clusters corresponds to the first simulation scenario.

well. Two samples from clusters 4, however, formed a small cluster relatively deep in the tree, and they appear to be more similar to that of the cluster 3. Still, the piecewise snipping (red rectangles) discerns them from the 3-cluster and identifies all the known clusters (ARI = 1). The fixed-height cut (blue broken line), however, is forced to merge the 3-cluster with the two samples from true cluster 4, thus produced a sub-optimal result (ARI= 0.93).

- The scenario where a HC tree has somewhat heterogeneous nested clusters, with intermediate level of overlap ($BarOmega = 0.01$).

  Although many distinct clusters were present deep in the HC tree (Additional file 1: Figure S3), the fixed-height cut produced three big clusters (ARI = 0.27) in this simulation. This is mainly due to the fact that the clusters were overlapping. The piecewise snipping, however, recovered many more of the true clusters (ARI = 0.46). Clearly, both approaches were plagued by the cluster overlap, but the latter appears to be more robust than the former.

- The scenario where a HC tree has highly heterogeneous clusters ($BarOmega = 0.1$).

  This is the most extreme scenario, where no clear clustering structure is present in the HC tree (Additional file 1: Figure S4). As a result, both approaches performed

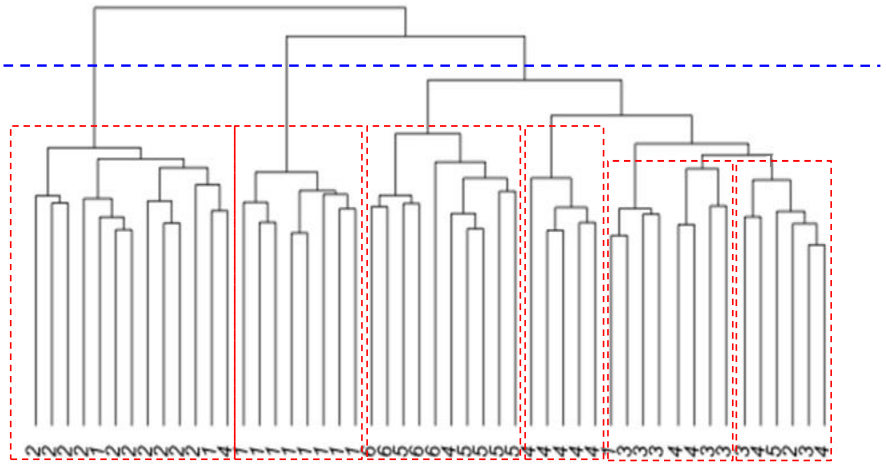

Additional file 1: Figure S3. A simulated HC tree with heterogeneous nested clusters corresponds to the second simulation scenario.

poorly, although the piecewise snipping produced a slightly larger ARI = 0.12 than fixed-height cut: ARI = 0.09. This scenario is meant to convey to message that at a bare minimum, the piecewise snipping still produces a clustering that is at least as good as fixed-height cutting. Here, poor performance indicates that the data and the resulting HC tree simply do not have a meaningful structure.

The HC tree snipping with the Dynamic Tree Cut

Here, we select the Dynamic Tree Cut algorithm (DTC) (Langfelder and Zhang, 2009) as a representative to highlight the problems in existing approaches. For this illustration we use the Lung cancer data (Beer *et al.,* 2002). Except for the argument "minClusterSize = 4", we use the default settings for the rest. The clusters generated from the DTC are shown in Additional file 1: Figure S5 along with the one from the fixed-height cut. The partition from the DTC renders $AIC = 204$ that is worse than the one from the piecewise snipping (see Figure 1). Although the DTC addresses the problem in the fixed-height cut by cutting at the variable heights, lack of guidance during the HC tree snipping process leads to a clustering that encompasses no or less association with that of clinical outcome.

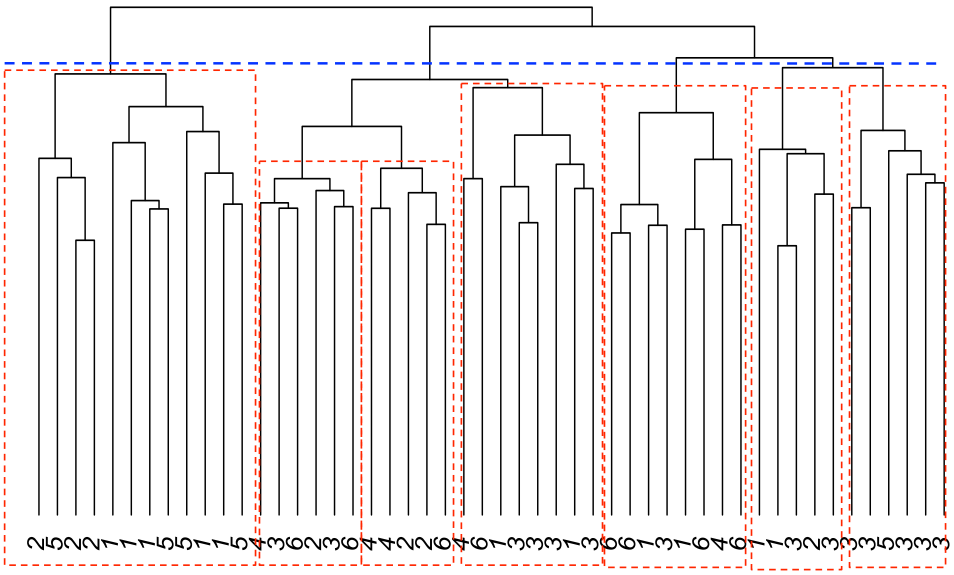

Additional file 1: Figure S4. A simulated HC tree with highly heterogeneous clusters corresponds to the third simulation scenario.

Differences between the piecewise snipping and exiting approach

The piecewise snipping approach we proposed here differs from the HC clustering method proposed by Dotan-Cohen *et al.* (2007) in the following regards:

- The latter is designed for gene clustering; application to clustering samples (tissues/disease/patients), however, is not straightforward. As emphasized by Baya and Granitto (2011) “clustering samples is very different from clustering genes”. The former, on the other hand, is designed for clustering samples in which the choice of data types is open .

- The latter cuts the HC tree at any place as long as resulting clusters are maximally consistent with pre-defined gene labels. This implies that the HC tree structure is allowed to change dramatically. We believe that a HC tree expresses the tendency of observations to be clustered by their signatures in the data from which the HC tree is derived. Although the resulting clusters are homogeneous in terms of partially available observation labels, this comes at the cost of losing its “honesty” to the data set the HC is based upon. To preserve the HC tree structure, the former selects a cut so that only the observations that are neighbours in the leaf nodes allowed to

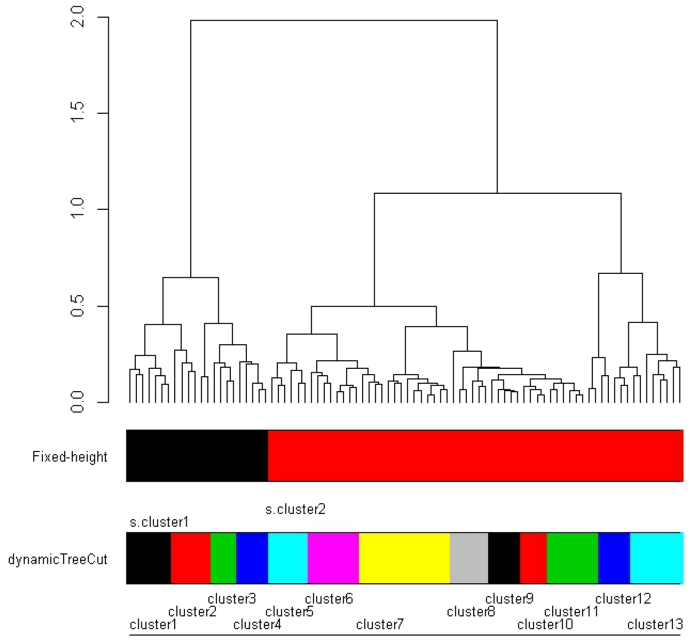

Additional file 1: Figure S5. The HC tree derived from the expression data from Beer *et al.,* (2002), and the cuts induced by the two approaches.

form clusters. For example, in Figure 2 the samples in *p.cluster1* are not allowed to form a cluster with the samples in *p.cluster3*.

- The latter only works with background information in discretized format. The former, however, has no restriction on the format of background information. To the best of our knowledge, we are the first to implement a HC tree snipping scheme which uses any types of data as a background information, that includes commonly available, clinically most relevant patient follow-up data.

Partition extraction from the HC tree

Here, we detail our implementation for extracting a compete set of partitions from a given HC tree. Say we are given a HC tree as in Additional file 1: Figure S6. We begin by spliting the HC tree into two branches (two clusters). Then, in each branch choose all existing pairs (black) and their possible augmented forms (blue). The result will be:
From the left branch :

$$\begin{array}{llll} \{4,6\} & \{4,6,7,8,1,5\}, & & \\ \{1,5\} & \{1,5,8\} & \{1,5,8,7\} & \{1,5,8,7,4,6\}. \end{array}$$

From the right branch:

$$\begin{array}{ll} \{3,10\} & \{3,10,2,9\}, \\ \{2,9\} & \{2,9,3,10\}. \end{array}$$

Note that, above shown pairs and their augmented forms are not made arbitrarily. We only take those that are made possible by the HC algorithm itself. For example, the

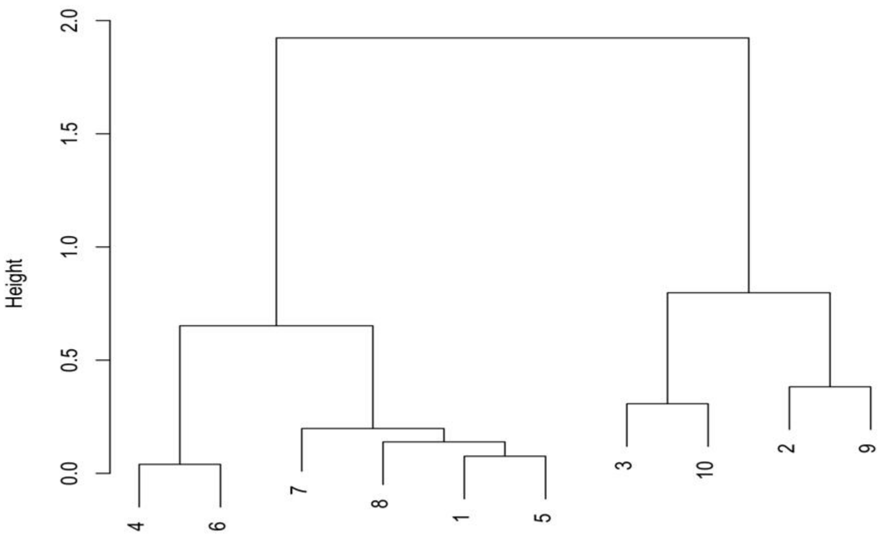

Additional file 1: Figure S6. An example HC tree.

following pairs are not allowed: (3, 2) and (10, 9). Combinations are made under the condition that, the HC tree structure should be preserved. Hence, the extracted clusters are faithful to the data set from which the HC is derived. Since we do not allow for singletons, we permit one exception to this rule: if a snip creates singleton(s), then these are assigned to the closest cluster that did not involve the particular snip. For example, in the example above, if we snip the $\{7, 8, 1, 5\}$ cluster just above '8', then $\{7\}$ would be merged with $\{4, 6\}$ to form $\{4, 6, 7\}$.

Select a pair (blue) from the right branch (the rest of the samples in this branch are merged to form a cluster), make all possible groupings with the pairs or their augmented forms on the left branch. Augment the selected pair, repeat the same grouping with clusters from the left branch. Repeat the same process for all the pairs on the right branch and delete the duplicated ones, then we will end up with the following set of unique partitions from this HC tree:

$\{3, 10\}$	$\{2, 9\}$	$\{4, 6\}$	$\{7, 8, 1, 5\}$	
$\{3, 10\}$	$\{2, 9\}$	$\{4, 6, 7, 8, 1, 5\}$		
$\{3, 10\}$	$\{2, 9\}$	$\{1, 5\}$	$\{4, 6, 7, 8\}$	
$\{3, 10\}$	$\{2, 9\}$	$\{1, 5, 8\}$	$\{4, 6, 7\}$	
$\{3, 10\}$	$\{2, 9\}$	$\{1, 5, 8, 7\}$	$\{4, 6\}$	
$\{3, 10, 2, 9\}$	$\{4, 6\}$	$\{7, 8, 1, 5\}$		
$\{3, 10, 2, 9\}$	$\{4, 6, 7, 8, 1, 5\}$			
$\{3, 10, 2, 9\}$	$\{1, 5\}$	$\{4, 6, 7, 8\}$		
$\{3, 10, 2, 9\}$	$\{1, 5, 8\}$	$\{4, 6, 7\}$		
$\{3, 10, 2, 9\}$	$\{1, 5, 8, 7\}$	$\{4, 6\}$		
$\{1, 5\}$	$\{7, 8\}$	$\{4, 6\}$	$\{3, 10\}$	$\{2, 9\}$
$\{1, 5\}$	$\{7, 8\}$	$\{4, 6\}$	$\{3, 10, 2, 9\}$	
$\{1, 5, 8\}$	$\{4, 6, 7\}$	$\{3, 10\}$	$\{2, 9\}$	
$\{1, 5, 8\}$	$\{4, 6, 7\}$	$\{3, 10, 2, 9\}$		

`PNN+Concordance`

Besides selecting the optimal partition in a semi-supervised way, we try to assign cluster labels to the test set in a semi-supervised way as well. To this end, we propose a novel approach called `PNN+Concordance`. It attempts to directly assess the similarity between the test and the training sets using their molecular profiles, and indirectly assess their similarity in terms of time-to-event information using the pseudo nearest neighbour (PNN) (Obulkasim *et al.* 2011). Here, we use a toy example to illustrate how `PNN+Concordance` works.

Say we have 10 samples in the training set, for which both gene expression data and time-to-event information are available. Going through the three steps mentioned in the paper, a partition with two clusters is selected as the optimal one. Suppose that cluster labels and time-to-event information of these 10 samples are:

$$\begin{aligned}&\text{cluster} = (1, 1, 1, 1, 1, 2, 2, 2, 2, 2),\\&\text{surv.time} = (2.6, 4.0, 4.5, 4.9, 5.4, 25.3, 26.8, 28.2, 28.3, 29.4),\\&\text{event} = (0, 1, 0, 0, 0, 1, 1, 1, 0, 0).\end{aligned}$$

A new sample arrives, for which only the gene expression profile is available. We project this new sample onto the expression data space and measure its similarity with each of the 10 training samples. Say after ordering (descending) the similarities, we obtain the following order indices:

$$\text{ord}^{\text{test}} = (10, 3, 6, 4, 1, 9, 5, 2, 7, 8).$$

Save the indices of the first $k = 5$ samples from the list (we set k to the number of samples in a cluster under consideration). Note that, both expression profiles and time-to-event information are available for these samples. We treat these samples as the pseudo nearest neighbours (PNN) of the new sample in the patient time-to-event data space. The logic behind this is that when two samples have similar molecular characteristics, then they may also share clinical characteristics due to the potential association between the two types of features.

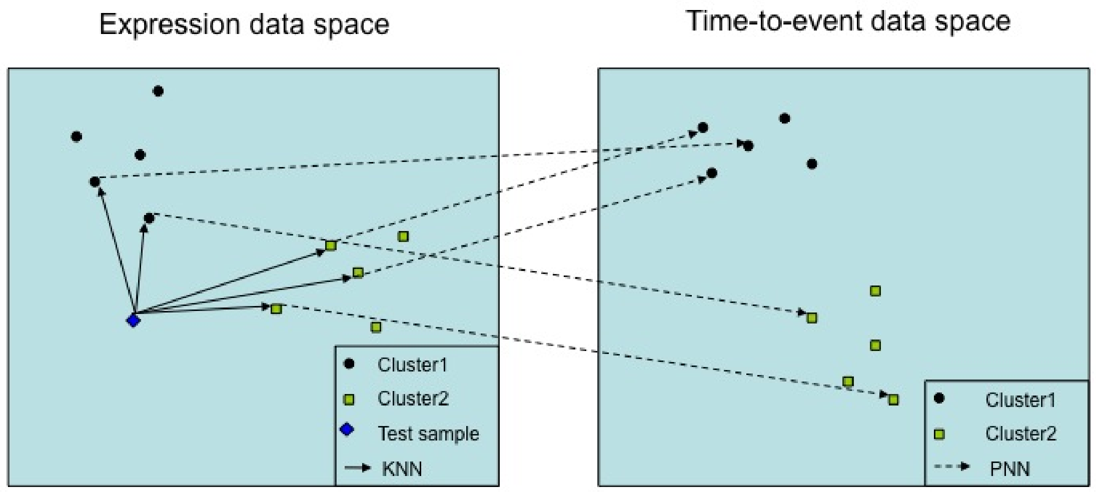

Additional file 1: Figure S7. Illustration of the expression to the time-to-event data space projection, $k = 5$.

Select the first sample from the PNN list, make all possible pair of cases with samples in the first cluster. The result is:

$$\begin{array}{ll} (29.4,\ 2.6) & (0,\ 0), \\ (29.4,\ 4.0) & (0,\ 1), \\ (29.4,\ 4.5) & (0,\ 0), \\ (29.4,\ 4.9) & (0,\ 0), \\ (29.4,\ 5.4) & (0,\ 0). \end{array}$$

Take the first pair (say a and b) from the pairing list, name them as the active set and the rest of the 8 samples (9 if the active set pair are the same) as the reference set (ω). Choose a sample from the reference set (reference sample ref) and combine it with the active set. Calculate the similarity of (a, b) based on the censoring status of these triplet using the Concordance index (Harrel *et al.* 1982) which is defined by:

1. If the reference sample has an event and both member of the active set are censored

$$\text{case}^1 = \sum_{i \in \omega} I\{t_a \geq t_i^{\text{ref}} \text{ AND } t_b \geq t_i^{\text{ref}}\}, \tag{1}$$

2. If the reference sample has an event and the active set also have events

$$\text{case}^2 = \sum_{i \in \omega} I\{(t_a \geq t_i^{\text{ref}} \text{ AND } t_b \geq t_i^{\text{ref}}) \text{ OR } (t_a < t_i^{\text{ref}} \text{ AND } t_b < t_i^{\text{ref}})\}, \tag{2}$$

3. If the reference sample is censored and the active set have events

$$\text{case}^3 = \sum_{i\in\omega} I\{t_a \le t_i^{\text{ref}} \text{ AND } t_b \le t_i^{\text{ref}}\}, \tag{3}$$

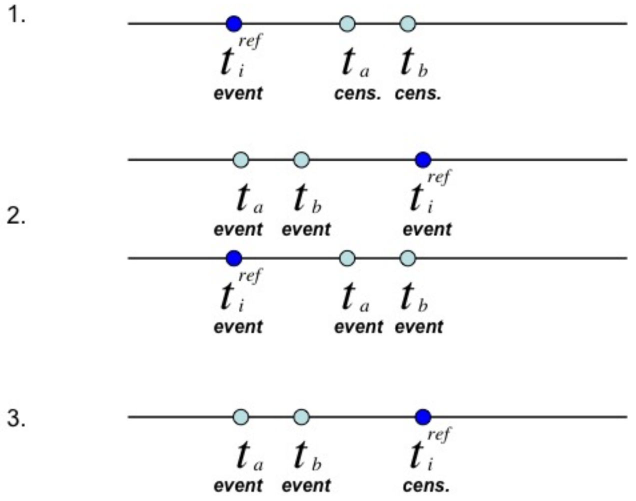

Additional file 1: Figure S8. Illustration of the three scenarios in the Concordance index calculation.

where $I\{\cdot\} = 1$ if the argument is true and $= 0$ otherwise, t_a is the survival time of the patient a, t_i^{ref} is the survival time of the ith reference sample. Finally, the similarity of the pair (a, b) is equal

$$\text{C-index}_{a,b} = \frac{\sum_{j=1}^{3} \text{case}^j}{|\Phi|}, \tag{4}$$

where Φ is the set that consists of all triplets that satisfies one of the aforementioned conditions. C-index ranges from 0 to 1, with 1 denoting a perfect match.

Following above calculation steps, we obtain the following similarities between each of the test sample's PNNs and the existing two clusters

**PNN**	**cluster1**	**cluster2**
10	0.357	0.516
3	0.727	0.667
6	0.571	0.08
4	0.727	0.667
1	0.000	0.000

Weight each the similarity with the inverse of the ordering index. For the cluster1 we obtain

$$\frac{0.357}{1} + \frac{0.727}{2} + \frac{0.571}{3} + \frac{0.727}{4} + \frac{0}{5} = 1.093. \tag{5}$$

Analogously, we obtain 0.989 for the cluster2. Thus, `PNN+Concordance` assigns the new sample to the first cluster.

Note that, the value of the k varies according to cluster size. This may bias towards the big cluster. One may re-weight the similarities according to cluster size. We dot not do so because, 1) We apply the inverse weighting scheme. This way of weighting diminish the effect of a neighbour located far from the new sample. Thus, large k (which equals more summation terms in (5)) does not effect much on the final similarity. 2) The prior probability for a new sample to be assigned to a large cluster should be somewhat larger than that of a small cluster.

Clustering on the new samples on the presence of multiple HC trees

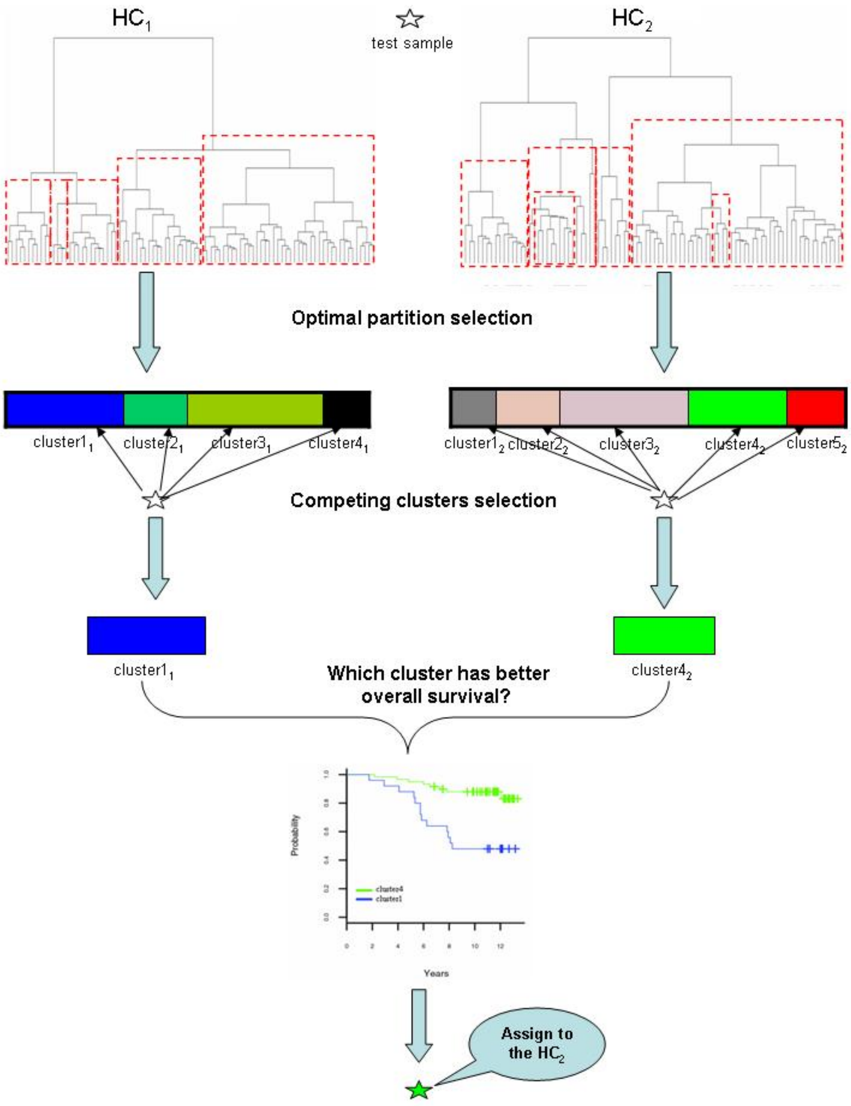

Additional file 1: Figure S9. The workflow of assigning new sample to given HC trees.

The piecewise snipping vs. the fixed-height cut approach: results not included in the paper

Note that, except for the C-index, we use the R-package **fpc** (Hennig, 2010) to calculate WSS and GK measures. For a given partition and corresponding distance matrix, *cluster.stats* function in this package returns a overall quality score for it.

Additional file 1: Table S2. Comparison of the error rates from the two approaches when the C-index is used for the gene expression data. In each column, numbers denote the number of times our method produces smaller error rates than the fixed-height cut in 100 repetitions, and the opposite holds for numbers in parentheses.

**Data**	**Ward**		**PNN+Concordance**	
	AIC	*BIC*	*AIC*	*BIC*
Lung.1	61(32)	61(33)	15(8)	15(9)
Leukemia	67(33)	73(27)	81(19)	91(7)
Lung.2	65(35)	62(37)	74(22)	74(20)
Lymphoma	57(43)	66(34)	68(31)	82(18)
Prostate	45(54)	51(49)	65(34)	72(28)
Glioblastoma	67(33)	73(27)	54(44)	65(35)

Additional file 1: Table S3. Comparison of the error rates from the two approaches when the GK is used for the gene expression data. In each column, numbers denote the number of times our method produces smaller error rates than the fixed-height cut in 100 repetitions, and the opposite holds for numbers in parentheses.

**Data**	**Ward**		**PNN+Concordance**	
	AIC	*BIC*	*AIC*	*BIC*
Lung.1	63(31)	63(30)	14(12)	15(16)
Leukemia	71(27)	70(28)	74(24)	79(16)
Lung.2	53(47)	51(49)	74(20)	68(24)
Lymphoma	61(38)	62(37)	65(32)	79(20)
Prostate	44(49)	42(41)	52(39)	53(29)
Glioblastoma	46(42)	52(19)	45(42)	42(28)

Association between the optimal clustering found in the Lung.1 data set and external clinical outcomes

In our analysis, we used the patient follow-up information in the cluster extraction process. To make the comparison between the piecewise snipping and the fixed-height cut methods thoroughly enough, we go one step further to check the association between the optimal partitions retrieved by the two approaches and the clinical outcomes not used in the cluster extraction process. For this illustration, again, we used the Lung.1 data set.

Besides the follow-up data, other clinical information such as disease stage and differentiation were also available for this data set. Chi-square test is used to check the association between the aforementioned two clinical variables and the the best partitions

found when the different quality measures are used. Results are given in Additional file 1: Table S3.

Additional file 1: Table S4. Association between the optimal partitions generated by the piecewise snipping and the fixed-height (in parentheses) cut with the external clinical outcomes.

Method		Stage	Differentiation
	WSS	0.052(0.859)	0.348(1)
**AIC with**	C-index	0.051(0.859)	0.434(1)
	GK	0.002(0.859)	0.674(1)
	WSS	0.052(0.924)	0.373(1)
**BIC with**	C-index	0.053(0.924)	0.517(1)
	GK	0.002(0.924)	0.690(1)

Regardless of which cluster quality measures being used, the fixed-height cut approach generates the same result (a partition with the two clusters). The piecewise snipping, on the other hand, produces slightly different results in different criteria settings. In all cases, the best partitions selected by the fixed-height approach exhibits no association with Stage or Differentiation. While the optimal partitions from the piecewise snipping exhibit weak associations with the Differentiation, strong associations with the Stage are observed. These results further show the potential of the piecewise snipping. Note that the p-values we present here are bias, because we did not re-snip the HC tree under permutation. The potential bias caused by the follow-up information which we used during the cluster extraction process is correlated with the two new clinical outcomes we tested here. Here, we only mean to show the differences in the magnitude of the p-values from the two approaches.

Logrank test p-value calculation by permutation

When studying association of cluster membership with clinical follow-up, such as survival data, we cannot use the standard testing procedures when our semi-supervised approach has been applied: we would use the follow-up data twice and the resulting p-value is likely to be too small. We avoid this bias by also applying the semi-supervised cluster construction under the null-hypothesis. This null-hypothesis is simply the absence of association between the samples' molecular information and the follow-up. Then, our cluster construction in combination with a suitable test statistic is designed to detect associations that can be represented by groups of samples. We adapt the p-value computation as follows.

1. For the observed data, compute the clusters using a semi-supervised approach (piecewise or fixed-height cut)
2. Use a suitable test statistic (e.g. log-rank for time-to-event data) to compute the *conditional* p-value given the resulting clusters: p_{obs}.
3. For $i = 1, \ldots, B$ (e.g. $B = 10,000$):
   (a) Randomly permute the follow-up data across the samples.
   (b) Apply exactly the same semi-supervised approach for each instance.

(c) Conditional on the resulting clusters, compute p-value p_i.

4. Finally, compute the p-value of interest: $p = P(p_{\text{obs}} \geq p_i) = (\#i : p_{\text{obs}} \geq p_i)/B$.

Here, p satisfies a crucial property of p-values: it is uniformly distributed when the null-hypothesis is true, because p_{obs} and p_i are exchangeable random variables. The exchangeability is a result from the null-hypothesis and the use of exactly the same procedures to compute p_{obs} and p_i.

Visualization of the optimal cuts induced by the two approaches and survival curves of the resulting clusters

Here, we present the HC trees and the cuts induced by the two approaches not included in the paper. In all illustrations the BIC criteria is used for the follow-up data, and the KG criteria is used for the expression data. In each HC tree, the blue broken line denotes the cut induced by the fixed-height cut approach, and the red rectangles corresponds to the cuts from the piecewise snipping. Numbers in leaf node denote the survival time and the event status indicator (0 or 1), respectively.

- The leukemia data set (Bullinger *et al.* 2004)

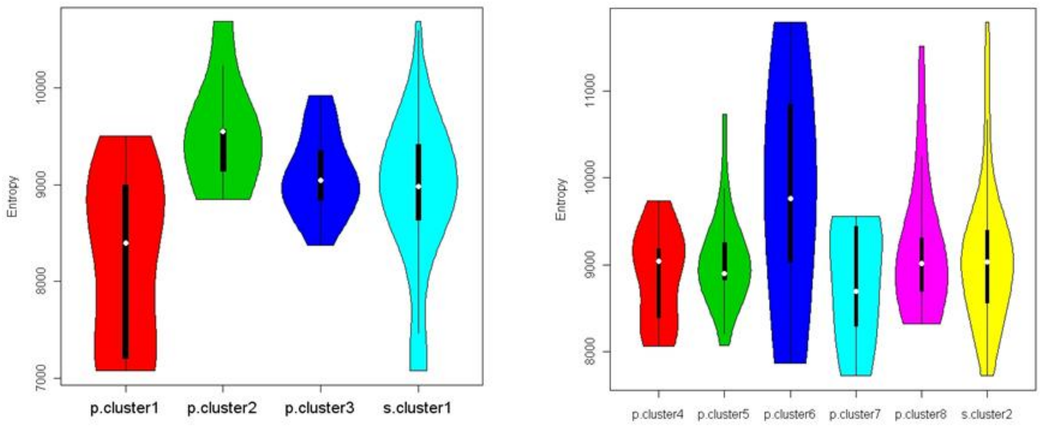

Additional file 1: Figure S10. The left panel plot shows the entropy distributions in the clusters on the left branch of the HC tree in Figure 4. The right panel corresponds to the clusters on the left branch. Entropy is a measure of inter-group variation (van Wieringen, 2010).

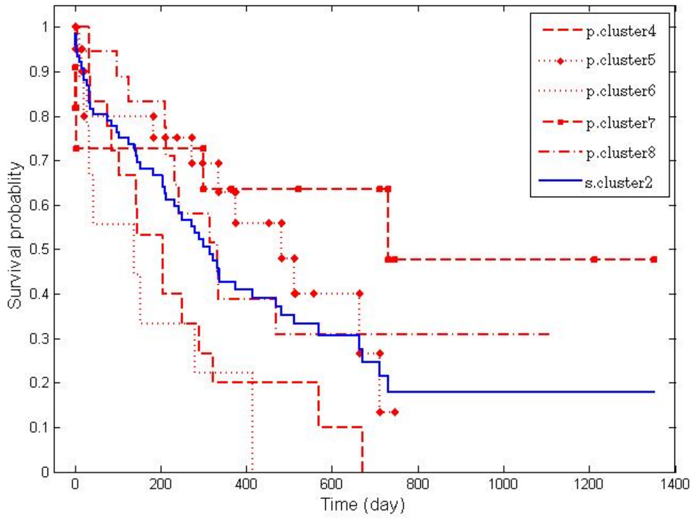

Additional file 1: Figure S11. The survival curves correspond to the clusters discovered on the right branch of the HC tree in Additional file 1: Figure 4.

- The Lung.1 data set (Beer *et al.* 2002)

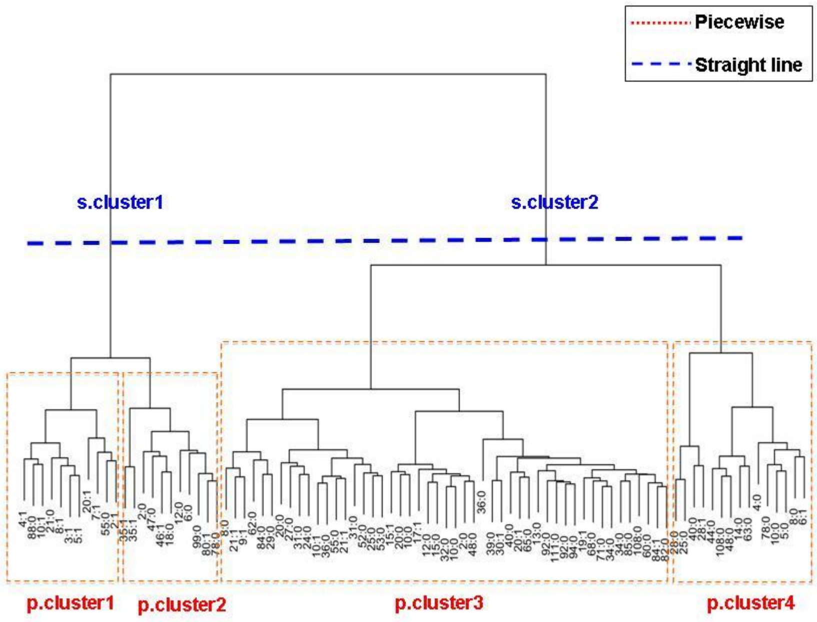

Additional file 1: Figure S12. The HC tree derived from the Lung.1 data set.

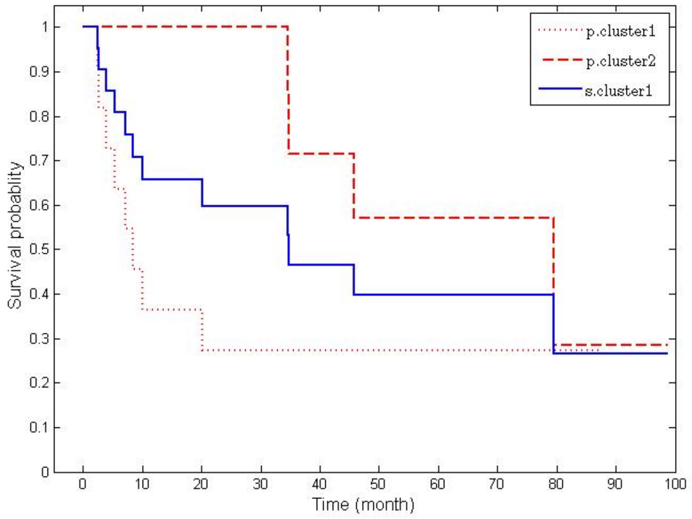

Additional file 1: Figure S13. Survival curves correspond to the clusters discovered on the left branch of the HC tree in Additional file 1: Figure S12.

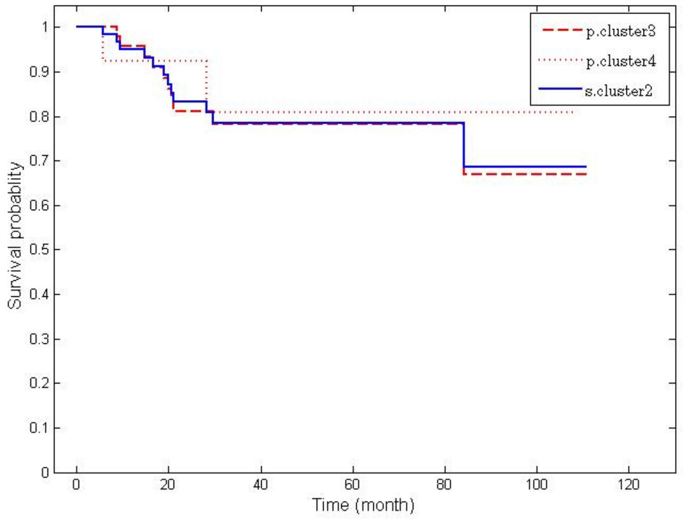

Additional file 1: Figure S14. Survival curves correspond to the clusters discovered on the right branch of the HC tree in Additional file 1: Figure S12.

- Lung.2 data set (Bhattacharjee *et al.* 2001)

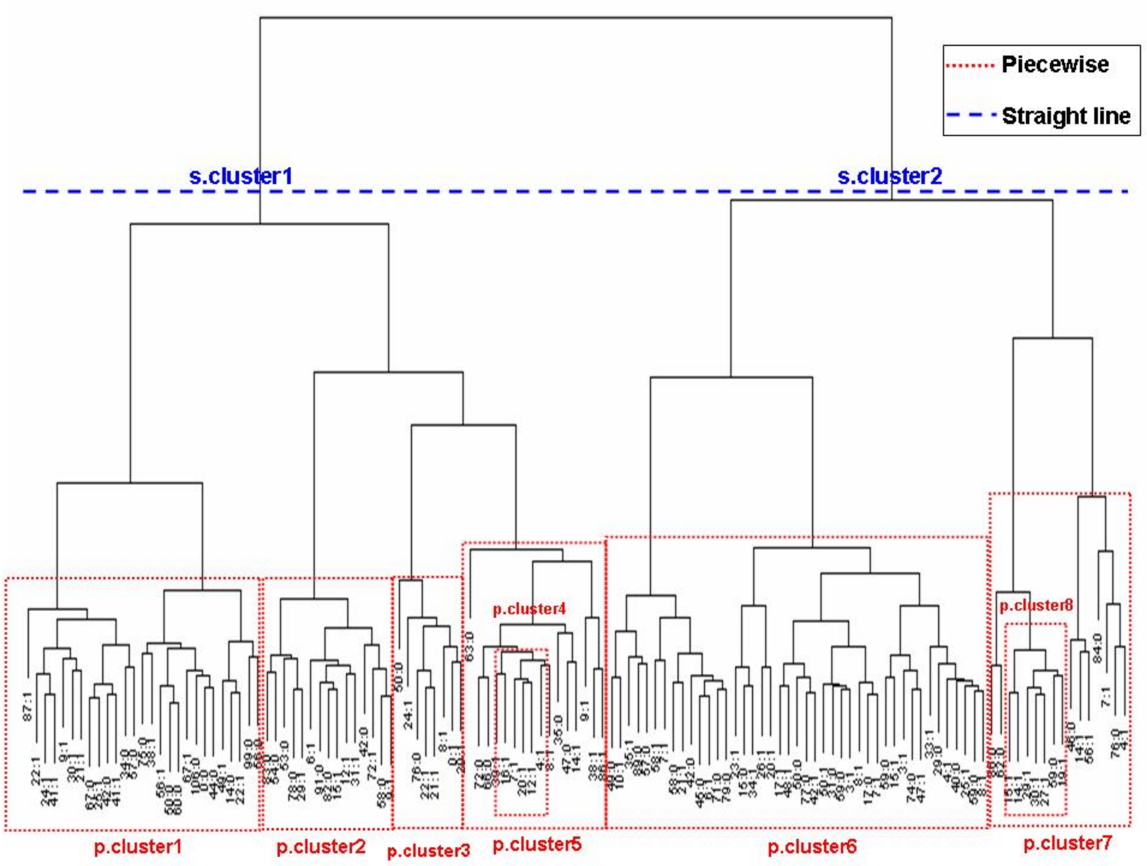

Additional file 1: Figure S15. The HC tree derived from the Lung.2 data set.

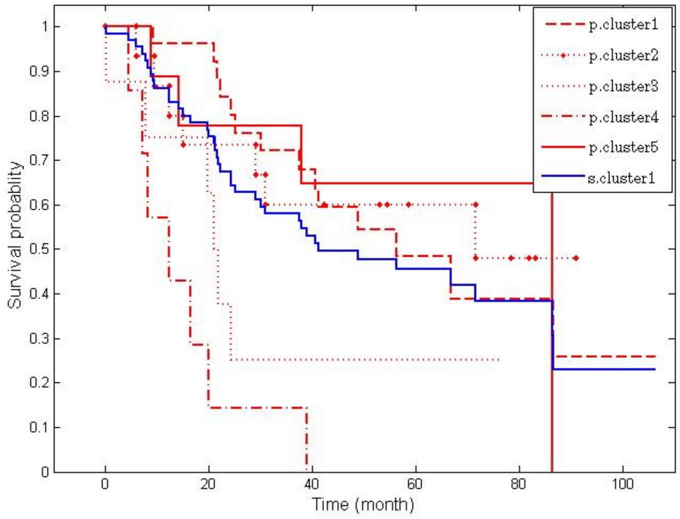

Additional file 1: Figure S16. Survival curves correspond to the clusters discovered on the left branch of the HC tree in Additional file 1: Figure S15.

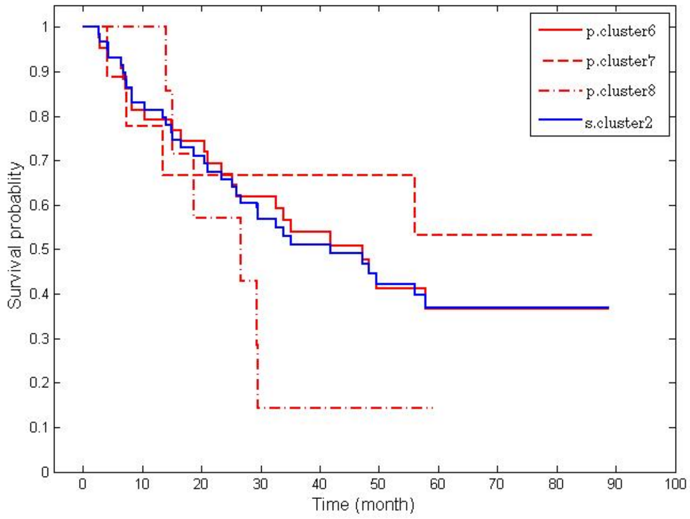

Additional file 1: Figure S17. Survival curves correspond to the clusters discovered on the right branch of the HC tree in Additional file 1: Figure S15.

- Lymphoma data set (Rosenwald *et al.* 2002)

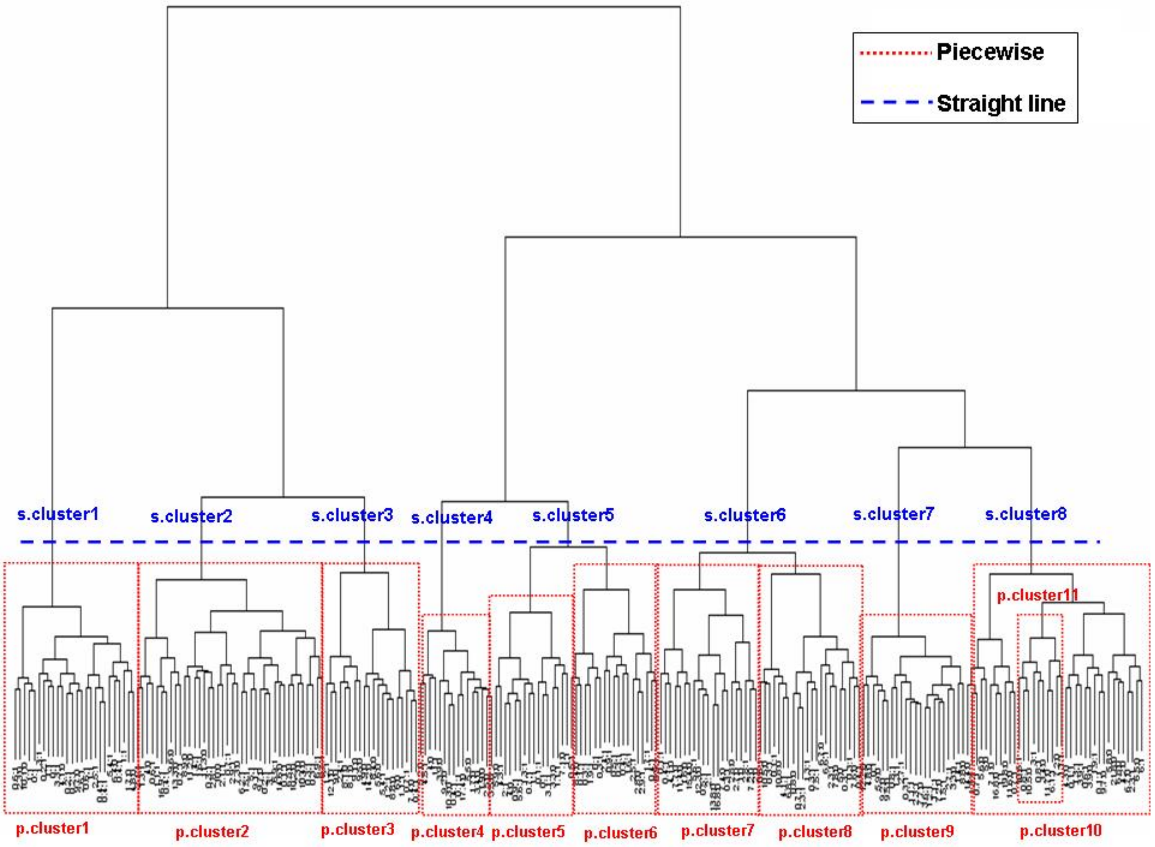

Additional file 1: Figure S18. The HC tree derived from the Lymphoma data set.

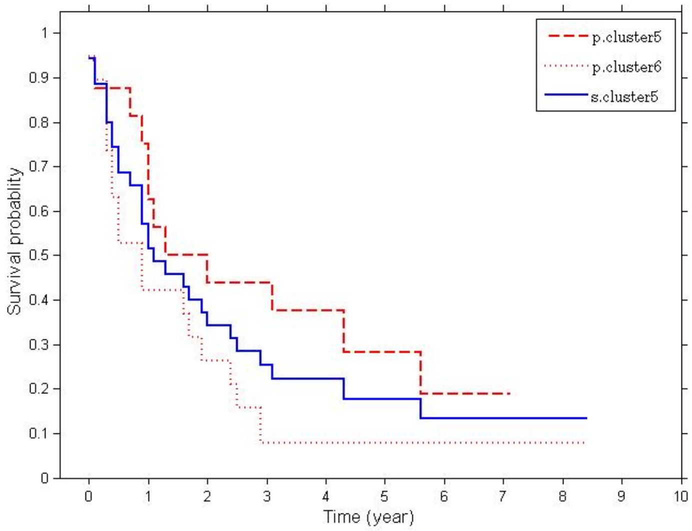

Additional file 1: Figure S19. Survival curves correspond to the *s.cluster5*, *p.cluster5-6* in Additional file 1: Figure S18.

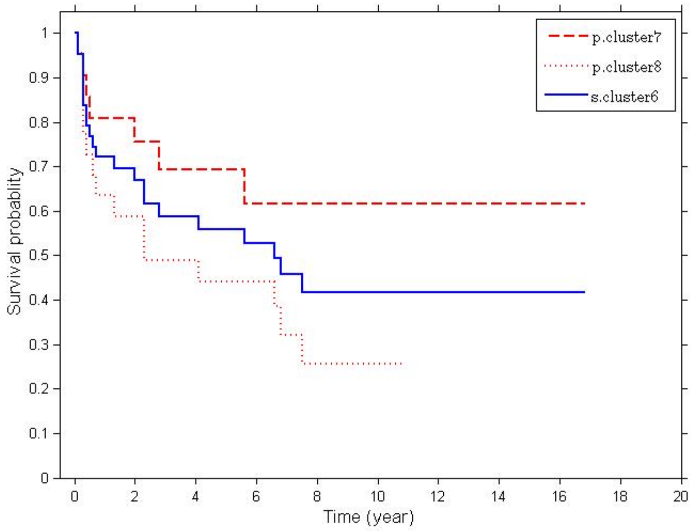

Additional file 1: Figure S20. Survival curves correspond to the *s.cluster6*, *p.cluster7-8* in Additional file 1: Figure S18.

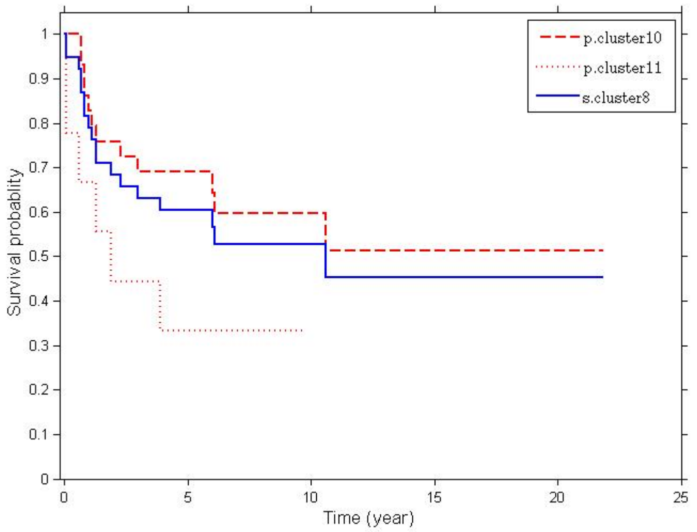

Additional file 1: Figure S21. Survival curves correspond to the *s.cluster8*, *p.cluster10-11* in Additional file 1: Figure S18.

- Prostate cancer data set (Sboner *et al.* 2010)

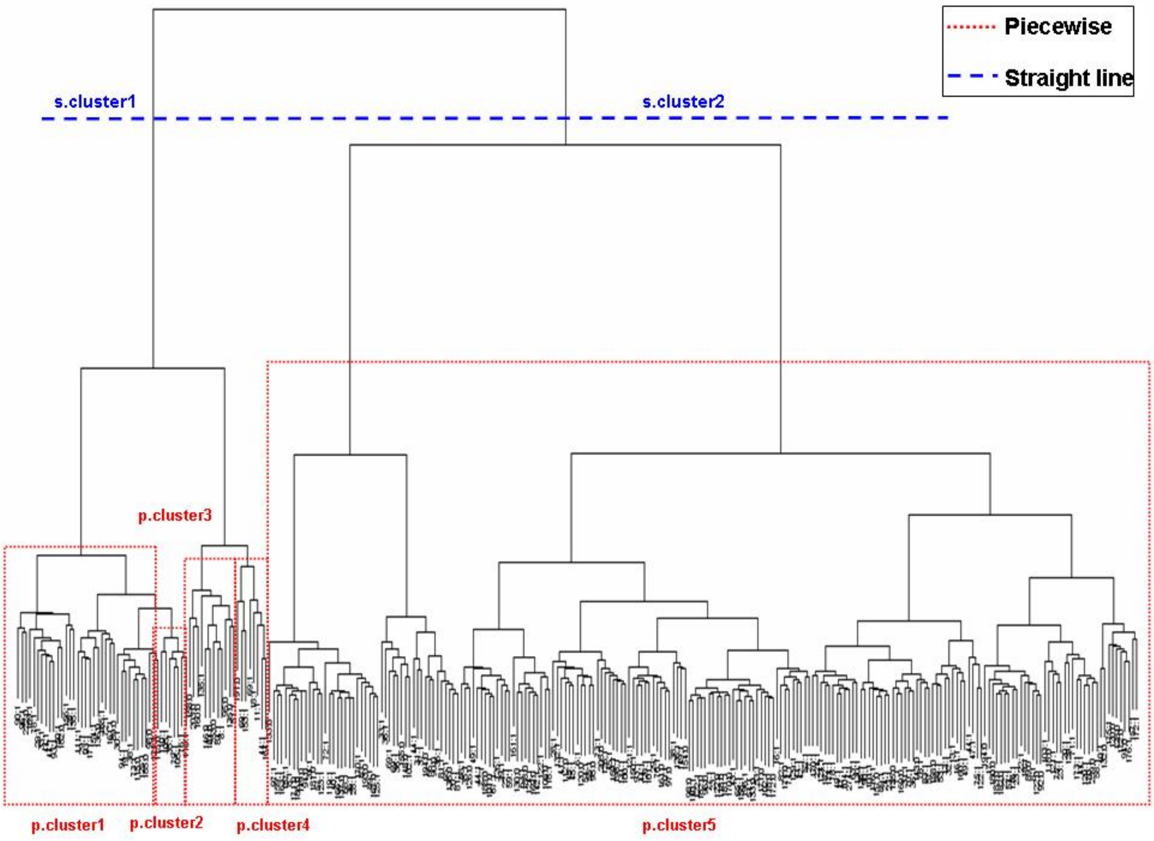

Additional file 1: Figure S22. The HC tree derived from the Prostate cancer data set.

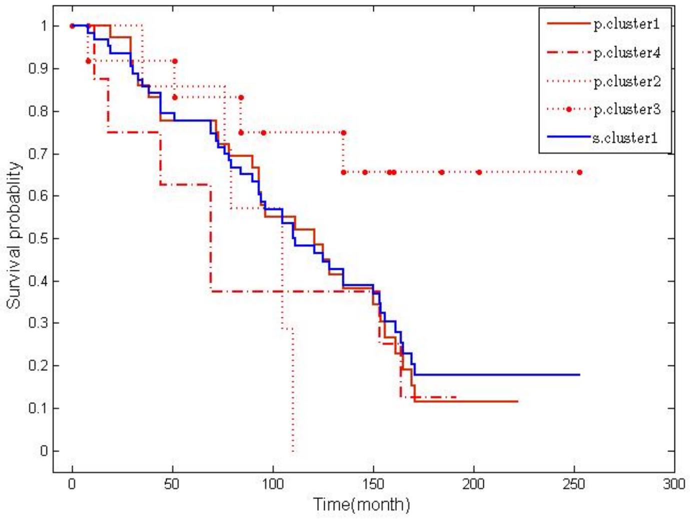

Additional file 1: Figure S23. Survival curves correspond to the *s.cluster1*, *p.cluster1-4* in Additional file 1: Figure S22.

- Glioblastoma data set (Verhaak *et al.* 2010)

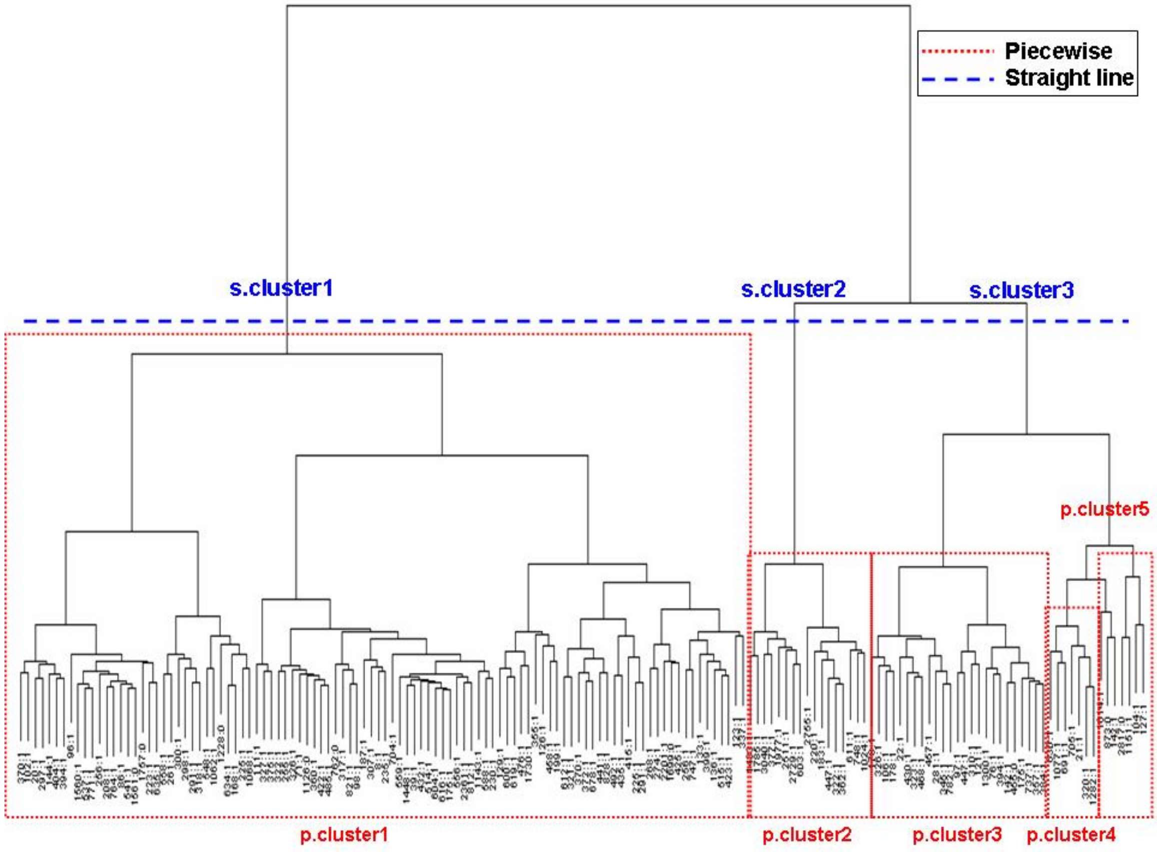

Additional file 1:Figure S24. The HC tree derived from the Glioblastoma data set.

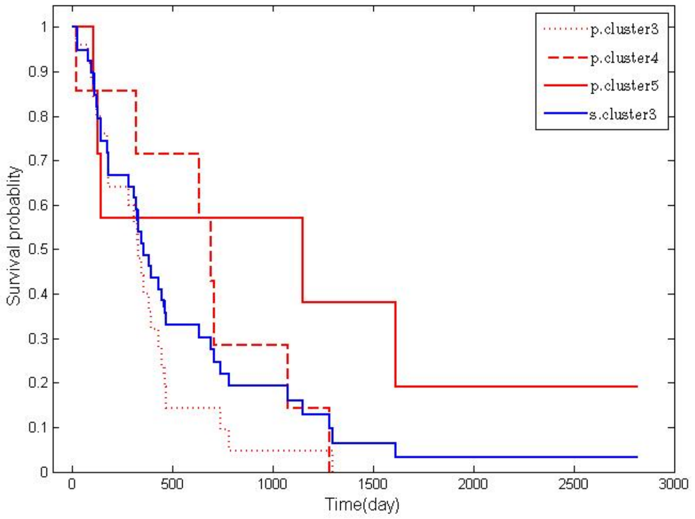

Additional file 1: Figure S25. Survival curves correspond to the *s.cluster2*, *p.cluster3-5* in Additional file 1: Figure S24.

References

1 Sørlie T *et al.* (2001) Gene expression patterns of breast carcinomas distinguish tumor subclasses with clinical implications, *Proc Natl Acad Sci USA*, **98**, 10869-10874.

2 Melnykov V *et al.* (2012) MixSim: An R package for simulating data to study performance of clustering algorithms, *Journal of Statistical Software*, **51**, 1-25.

3 Langfelder P and Zhang B (2009) Methods for detection of clusters in hierarchical clustering dendrograms, *R package*, http://cran.r-project.org/web/packages/dynamicTreeCut/.

4 Dotan-Cohen D *et al.* (2007) Hierarchical tree snipping: clustering guided by prior knowledge, *Bioinformatics*, **23**, 3335-3342.

5 Baya EA and Granitto MP (2011) Clustering gene expression data with a penalized graph-based metric, *BMC Bioinformatics*, **12**.

6 Obulkasim A *et al.* (2011) Stepwise classification of cancer samples using clinical and molecular data, *BMC Bioinformatics*, **12**, 422.

7 Harrel FE *et al.* (1982) Evaluating the yield of medical tests, *JAMA*, **247**, 2543-2546.

8 Hennig C (2010) Flexible procedures for clustering, *R package*, http://CRAN.R-project.org/package=fpc.

9 van Wieringen WN and van der Vaart AW (2010) Statistical analysis of the cancer cell's molecular entropy using high-throughput data, *Bioinformatics*, **27**, 556-563.

10 Beer DG *et al* (2002) Gene-expression profiles predict survival of patients with lung adenocarcinoma, *Nature*, **8**, 816-824.

11 Bhattacharjee A *et al.* (2001) Classification of human lung carcinomas by mRNA expression profiling reveals distinct adenocarcinoma subclasses, *Proc Natl Acad Sci USA*, **98**, 13790-13795.

12 Rosenwald L *et al.* (2002) The use of molecular profiling to predict survival after chemotherapy for diffuse large-B-cell lymphoma, *N Engl J Med.*, **346**, 1937-1947.

13 Sboner A *et al.* (2010) Molecular sampling of prostate cancer: a dilemma for predicting disease progression, *BMC Med Genomics*, **3**, 8-19.

14 Verhaak GWR *et al.* (2010) Integrated genomic analysis identifies clinically relevant subtypes of glioblastoma characterized by abnormalities in PDGFRA, IDH1, EGFR, and NF1, *Cancer Cell*, **17**, 98-110.

15 Yu T *et al.* (2011) Adjusting confounders in ranking biomarkers: a model-based ROC approach, *Brief Bioinform*, **13**, 513-523.
